# The CRISPR-Cas System Differentially Regulates Surface-Attached and Pellicle Biofilm in Salmonella enterica Serovar Typhimurium

**DOI:** 10.1128/spectrum.00202-22

**Published:** 2022-06-09

**Authors:** Nandita Sharma, Ankita Das, Pujitha Raja, Sandhya Amol Marathe

**Affiliations:** a Department of Biological Sciences, Birla Institute of Technology and Science (BITS), Pilani, Rajasthan, India; Ohio State University

**Keywords:** *Salmonella*, type I-E CRISPR-Cas system, surface-attached biofilm, pellicle biofilm

## Abstract

The CRISPR-Cas mediated regulation of biofilm by Salmonella enterica serovar Typhimurium was investigated by deleting CRISPR-Cas components *ΔcrisprI*, *ΔcrisprII*, *ΔΔcrisprI crisprII*, and *Δcas op.* We determined that the system positively regulates surface biofilm while inhibiting pellicle biofilm formation. Results of real-time PCR suggest that the flagellar (*fliC*, *flgK*) and curli (*csgA*) genes were repressed in knockout strains, causing reduced surface biofilm. The mutants displayed altered pellicle biofilm architecture. They exhibited bacterial multilayers and a denser extracellular matrix with enhanced cellulose and less curli, ergo weaker pellicles than those of the wild type. The cellulose secretion was more in the knockout strains due to the upregulation of *bcsC*, which is necessary for cellulose export. We hypothesized that the secreted cellulose quickly integrates into the pellicle, leading to enhanced pellicular cellulose in the knockout strains. We determined that *crp* is upregulated in the knockout strains, thereby inhibiting the expression of *csgD* and, hence, also of *csgA* and *bcsA*. The conflicting upregulation of *bcsC*, the last gene of the *bcsABZC* operon, could be caused by independent regulation by the CRISPR-Cas system owing to a partial match between the CRISPR spacers and *bcsC* gene. The cAMP-regulated protein (CRP)-mediated regulation of the flagellar genes in the knockout strains was probably circumvented through the regulation of *yddx* governing the availability of the sigma factor σ^28^ that further regulates class 3 flagellar genes (*fliC*, *fljB*, and *flgK*). Additionally, the variations in the lipopolysaccharide (LPS) profile and expression of LPS-related genes (*rfaC*, *rfbG*, and *rfbI*) in knockout strains could also contribute to the altered pellicle architecture. Collectively, we establish that the CRISPR-Cas system differentially regulates the formation of surface-attached and pellicle biofilm.

**IMPORTANCE** In addition to being implicated in bacterial immunity and genome editing, the CRISPR-Cas system has recently been demonstrated to regulate endogenous gene expression and biofilm formation. While the function of individual *cas* genes in controlling Salmonella biofilm has been explored, the regulatory role of CRISPR arrays in biofilm is less studied. Moreover, studies have focused on the effects of the CRISPR-Cas system on surface-associated biofilms, and comprehensive studies on the impact of the system on pellicle biofilm remain an unexplored niche. We demonstrate that the CRISPR array and *cas* genes modulate the expression of various biofilm genes in Salmonella, whereby surface and pellicle biofilm formation is distinctively regulated.

## INTRODUCTION

The clustered regularly interspaced short palindromic repeats (CRISPR)-Cas system bestows adaptive immunity to bacteria against invading mobile genetic elements (MGE) (1). It captures protospacers from invading MGEs and incorporates them into the CRISPR array with the help of Cas proteins (2). The system has also been implicated in alternative functions like governing virulence and bacterial physiology (3). In some bacterial species, including Salmonella, selective protospacers have been found within the bacterial genome, thereby supporting the role of the CRISPR-Cas system in endogenous gene regulation (4, 5). Salmonella possesses a type I-E CRISPR-Cas system comprising two CRISPR arrays, CRISPR-I and CRISPR-II, and one *cas* operon (5). This system has been demonstrated to regulate biofilm formation in Salmonella enterica subspecies *enterica* serovar Enteritidis by regulating the quorum-sensing system (6). It also regulates the expression of outer membrane proteins in Salmonella enterica serovar Typhi, thereby impacting biofilm formation and resistance to bile (7).

Salmonella is one of the four leading causes of diarrheal diseases worldwide (8). Salmonellosis, a disease caused by Salmonella, presents a formidable threat to humans, while some serovars cause typhoid fever (9). Annually, ~14.3 million individuals suffer from typhoid fever, with 135,000 estimated deaths worldwide (10). Salmonella enterica forms biofilms on various surfaces, including medically important surfaces like medical devices (catheters, endoscopy tubes, etc.), as well as gallstones (11). This complicates the treatment processes. Biofilm formation on cholesterol-rich gallstones is conceived as a significant factor influencing the establishment of a chronic carrier state, accounting for 1 to 4% of total typhoid cases (12, 13). Biofilm aids Salmonella virulence by facilitating evasion of the host’s immune response and increasing antibiotic tolerance, as biofilms can be impenetrable to antibiotics (14). Salmonella biofilms are a concern in the food and packaging industries and act as a pathogen transmission source in food processing units (15, 16). Biofilms lead to Salmonella's persistence in the environment (17). Improper disinfection and cleaning leave behind food particles that act as the substrates for surface-attached biofilm formation (18). Environmental conditions like low temperature and pH in the food production chain favor biofilm formation (18). These biofilms are resistant to common disinfectants used in the food industry, thus safeguarding Salmonella throughout food processing (19, 20). This magnifies the problems and spread of infection.

Salmonella forms biofilm at the solid-liquid interface (bottom) and air-liquid interface (here called a pellicle biofilm [21]), depending on the nutritional status of the cells. It also forms a biofilm ring on the glass wall at the air-liquid interface (21) (here called a surface-attached biofilm). Different biofilm components play a crucial role in various types of biofilm formation (Table 1) (17, 21–24). Biofilm formation is intricately regulated by regulators (17) like cAMP-regulated protein (CRP), RpoS, CsgD, etc., in response to different environmental stimuli (osmolarity, nutrient availability, and host factors) (25).

**TABLE 1:** Conditions and critical biofilm components required for different biofilm types

Biofilm type	Required conditions in environment	Required conditions in laboratory	Major components	Temp (°C), incubation time
Surface-attached biofilm (ring) (21)	Medical devices, food processing units (24), standing and flowing water ecosystems (23)	Liquid media (LB, YESCA^*a*^) in microtiter wells, flow cells, or test tubes at static or dynamic conditions	Cellulose, LPS, curli, type III secretion apparatus, and flagella (17)	25–37, 12–24 h
Pellicle biofilm (21–23)	Stagnant water ecosystem, vinegar productions, drainage system (23)	Liquid YESCA media in tubes, flask, or microtiter wells under static conditions	Curli, LPS, and cellulose (23)	25–28, 3–4 days

a YESCA, yeast extract casamino acids.

Biofilm formation is a tightly regulated process requiring adhesins such as pili (26), flagella (26), and curli (27, 28) for substrate adhesion. Flagellum acts as a mechanosensor, triggering the surface-associated motility and polysaccharide synthesis (26), while curli is required for cell-cell cohesion (29), forming a biofilm monolayer. The monolayer is gradually embedded with extracellular polymeric substances (cellulose, curli, and LPS) (22), maturing into pellicle biofilm. The extracellular matrix constituents, like cellulose and curli, protect Salmonella against disinfectants (18). The pellicle biofilm may switch to bottom biofilm on a change in the nutritional status of cells (30). Depleting nutrients like amino acids triggers *csgD* expression that promotes bottom biofilm formation through increased expression of curli and cellulose (30).

This study evaluated if and how the endogenous CRISPR-Cas system regulates different biofilm phenotypes of Salmonella enterica subspecies *enterica* serovar Typhimurium (*S*. Typhimurium). We found that the CRISPR-Cas system differentially regulated surface-attached and pellicle biofilm formation by altering the expression of biofilm-associated genes.

## RESULTS

### CRISPR-Cas knockout strains show temporal variations in the biofilm formation.

**FIG 1:**
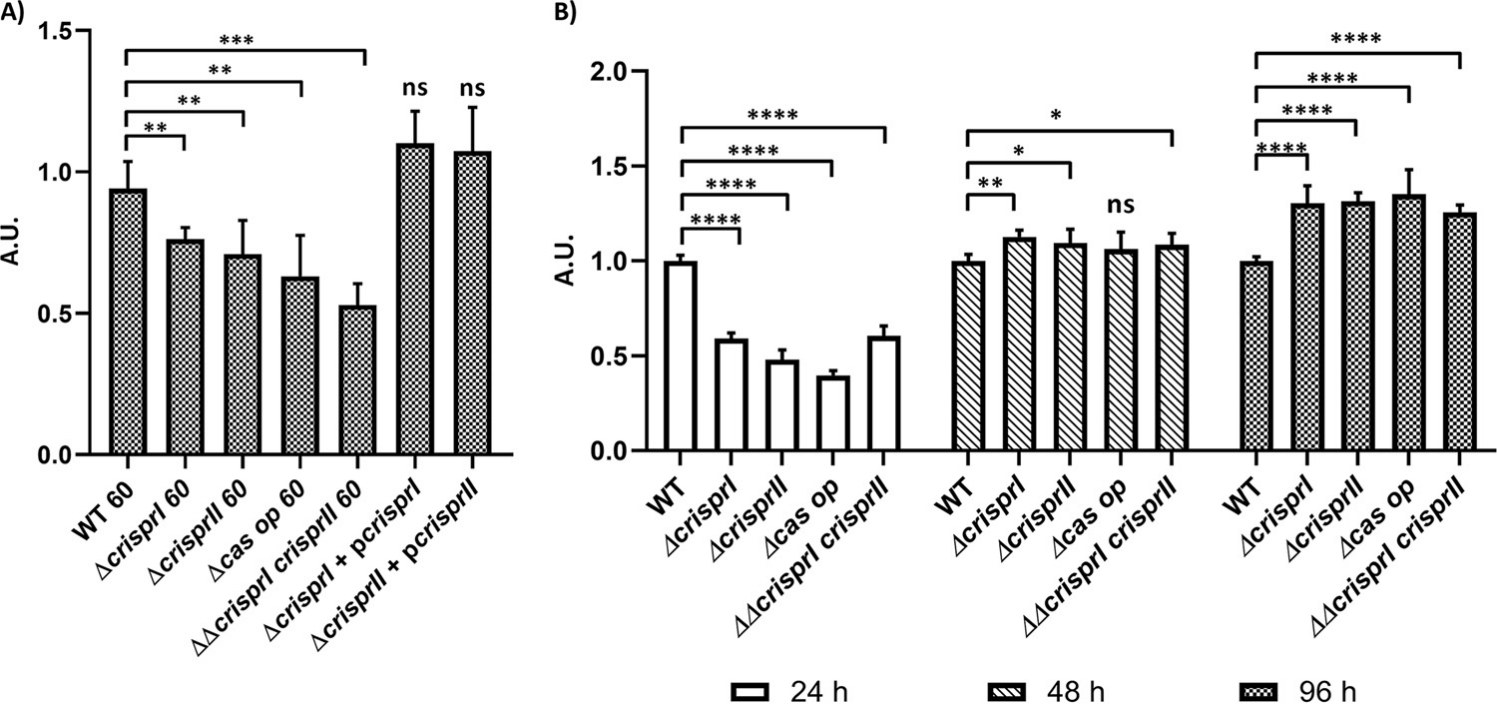
The CRISPR-Cas system knockout strains of S. enterica subsp. *enterica* serovar Typhimurium 14028s showed reduced biofilm formation under gallstone-mimicking conditions (A), while these strains showed temporal variations in biofilm at the solid-liquid-air interface (B). (A) Wild-type, CRISPR, and *cas* operon knockout strains transformed with empty vector pQE60 (WT60, *ΔcrisprI 60*, *ΔcrisprII 60*, *Δcas op. 60*, and *ΔΔcrisprI crisprII 60*), and the complement strains (*ΔcrisprI+*p*crisprI* and *ΔcrisprII+*p*crisprII*) were cultured in cholesterol-coated microcentrifuge tubes in LB media for 96 h at 37°C under static conditions. (B) *S.* Typhimurium strain 14028s wild-type (WT), CRISPR (*ΔcrisprI*, *ΔcrisprII*, and *ΔΔcrisprI crisprII*) and *cas* operon (*Δcas op.*) knockout strains were cultured in LB without NaCl media for different time periods (24 h, 48 h, and 96 h) at 25°C under static conditions. Biofilm formation was estimated using the crystal violet staining method. Graph represents optical density at 570 nm (OD_570_) for each strain, normalized by OD_570_ of WT. An unpaired *t* test was used to determine significant differences between the WT and knockout strains. Error bars indicate SD. Statistical significance is shown as follows: *, *P* ≤ 0.05; **, *P* ≤ 0.01; ***, *P* ≤ 0.001; ****, *P* < 0.0001; and ns, not significant. A.U., arbitrary units.

We tested the biofilm-forming ability of the CRISPR and *cas* operon knockout strains (*ΔcrisprI*, *ΔcrisprII*, *Δcas op.*, and *ΔΔcrisprI crisprII*) of *S*. Typhimurium strain 14028s under gallstone-mimicking conditions. For this purpose, cholesterol-coated tubes that create a uniform surface mimicking gallstones were used, and biofilm formation was tested in the presence of 3% ox bile (31). At the end of the 96 h, all the knockout strains showed reduced biofilm formation compared to wild type (WT) (Fig. 1A). The phenotypes exhibited by the knockout strains were restored on the complementation of corresponding genes in *ΔcrisprI* and *ΔcrisprII* (Fig. 1A). This outcome confirms that the gene deletions were clean without any side effects. Next, a time-dependent study determining the biofilm formation by the knockout strains in low osmotic conditions (LB without NaCl) showed temporal variations in biofilm phenotypes compared to that of the WT (Fig. 1B). The knockout strains formed a thin biofilm ring on the solid-liquid-air interface (surface biofilm) at 24 h (Fig. 1B) and 96 h (see Fig. S3A in the supplemental material).

However, as time progressed, the knockout strains displayed a gradual increase in the biofilm formation, with an ~1.3-fold increase in the biofilm at 96 h (Fig. 1B and Fig. S3B). The difference in observed biofilm phenotype was not accredited to the difference in bacterial growth, as testified by the similar growth patterns of all the strains in LB without NaCl media (Fig. S4).

### Scanning electron microscopy depicts the difference in biofilm architecture of CRISPR-Cas knockout strains.

Scanning electron microscopy (SEM) was used to investigate biofilm architecture at early (24 h) and late (96 h) time points. At 24 h, the micrographs of the WT showed more aggregated and tightly packed bacterial cells covering the large surface area (Fig. S5A). In contrast, the micrographs of all the knockout strains showed patchy bacterial aggregates (Fig. S5A).

Distinct bacterial cells were more evident in the *Δcas op*. strain than those of other Salmonella strains. Small dome-like structures were observed only in the WT micrograph, indicating the formation of the multilayered structure. The biofilm formed by the knockout strains displayed clumped cells without any slimy material in their vicinity. On average, all the strains had similar lengths at 24 h (Fig. S5B). However, a few elongated cells (marked in micrograph) were observed in the knockout strains at 24 h (Fig. S5A).

**FIG 2:**
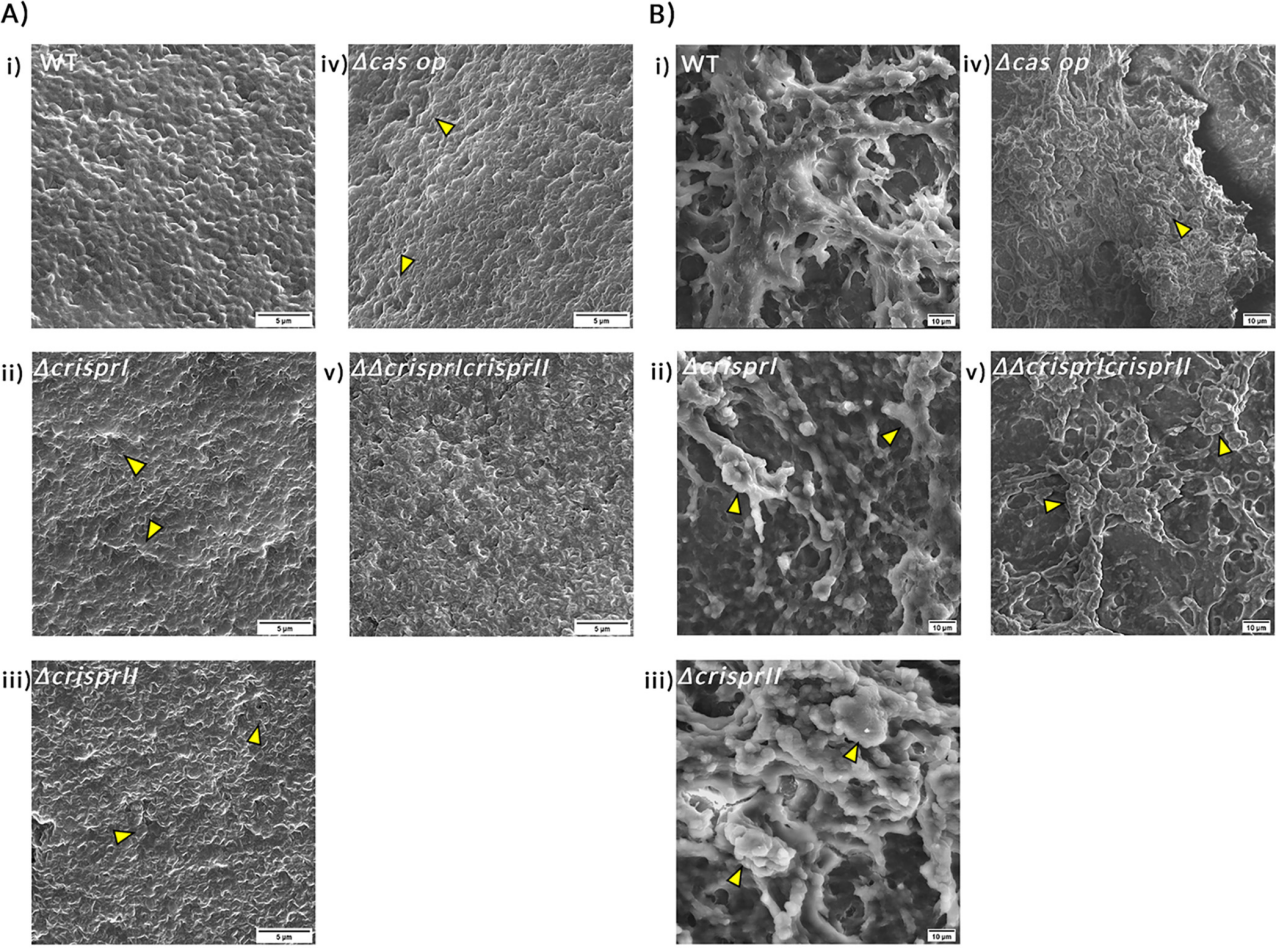
Morphology of the air-exposed side (A) and liquid-submerged side (B) of pellicle biofilm at 96 h. The strains were grown in LB without NaCl for 96 h at 25°C under static conditions. The pellicle biofilms were fixed using 2.5% glutaraldehyde and dehydrated with increasing ethanol concentrations. SEM image analysis depicts the difference in the pellicle biofilm architecture of CRISPR-Cas knockout (*ΔcrisprI*, *ΔcrisprII*, *Δcas op.*, *and ΔΔcrisprI crisprII*) strains and that of the wild type (WT) for both the air-exposed side (captured at ×10,000 magnification) and liquid-submerged side (captured at ×2,500 magnification) of pellicle biofilm. The air-exposed surface of the pellicle biofilm of CRISPR-Cas knockout strains had a denser, mat-like ECM. It consisted of “hilly” structures (marked with arrowheads), indicating more layering of the biofilm. The liquid-submerged surface of the pellicle biofilm of CRISPR-Cas knockout strains had more EPS lumps (marked with arrowheads) than the wild type. Images were scaled to bar.

SEM analysis of 96 h pellicle biofilm revealed that, in general, the air-exposed side of the pellicle biofilm had a dry but smooth mat-like structure composed of dense fibrous networks with tightly packed bacterial cells. However, compared to WT biofilm, the biofilms formed by knockout strains had thicker extracellular matrix (ECM) coatings and consisted of “hilly” structures of different sizes (Fig. 2A, arrowheads). The liquid-submerged side of the pellicle biofilm was rough, consisting of a dome- and valley-like arrangement made up of loosely packed bacterial cells entrapped in exopolymeric substances (EPS). The knockout strains also displayed discrete regions with EPS lumps (marked in micrographs) and pronounced bacterial density (Fig. 2B).

### Factors contributing to differential biofilm formation by CRISPR-Cas knockout strains.

To understand the knockout strains’ temporal variations in biofilm formation, we assessed the expression of essential biofilm components such as flagella, cellulose, LPS, and curli.

### (i) CRISPR-Cas knockout strains show reduced motility and flagellin expression.

**FIG 3:**
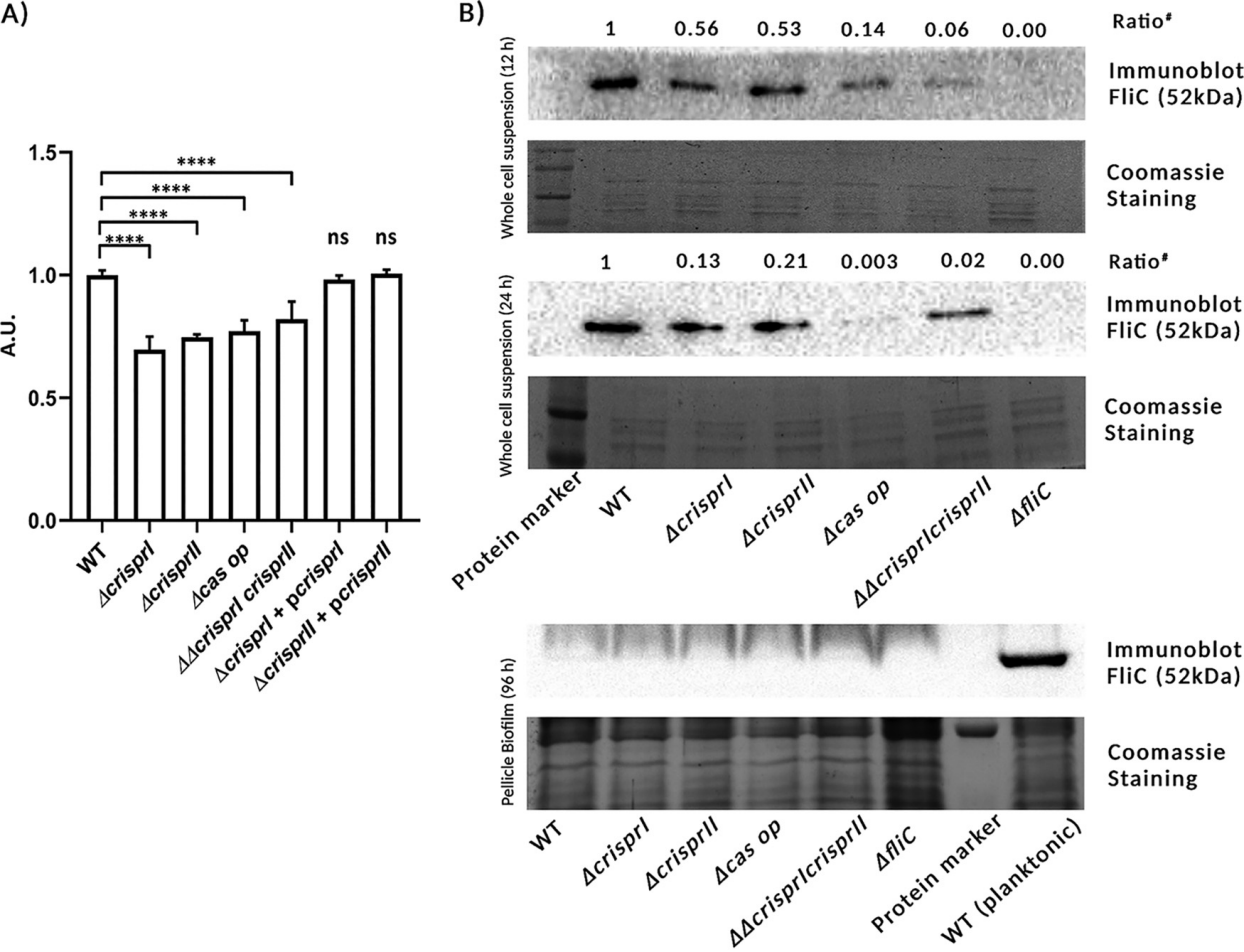
Reduced swarming motility (A) and expression of the flagellar protein, FliC (B), was observed in the CRISPR-Cas system knockout strains. (A) Overnight cultures were point inoculated on swarm agar plates and incubated at 37°C for 9 h. Swarming rates (cm/h) of the wild-type (WT) strain, the knockout strains (*ΔcrisprI*, *ΔcrisprII*, *Δcas op.*, and *ΔΔcrisprI crisprII*), and the complement strains (*ΔcrisprI* + p*crisprI*, *ΔcrisprII*+ p*crisprII*) were calculated. Graph represents the swarming rate relative to that of WT. (B) Strains were grown in LB without NaCl for different time periods (12 h, 24 h, and 96 h) at 25°C under static conditions. The expression of the flagellar protein in planktonic bacteria (B) at early time points (12 h and 24 h) and in pellicle biofilm (B) at a late time point (96 h) was assessed using Western blot analysis with antibodies against FliC. Even at higher protein concentration, FliC was not detected in the blot for pellicle sample of any strain, indicating repression of FliC expression in pellicle biofilm. *ΔfliC* was used as a negative control. An unpaired *t* test was used to determine significant differences between the WT and knockout strains. Error bar indicates SD. Statistical significance is represented as follows: *, *P* ≤ 0.05; **, *P* ≤ 0.01; ***, *P* ≤ 0.001; ****, *P* < 0.0001; ns, not significant. A.U., arbitrary units; #, ratio above the immunoblots (B), indicates the relative intensity of the bands with respect to wild type, observed in the blots, normalized by the relative intensity of the bands with respect to wild type, observed on the gel. The ratio is as follows: $\frac{{\left(\mathrm{FliC}\text{ intensity/Coomassie band intensity}\right)}_{\text{strain}}}{{\left(\mathrm{FliC}\text{ intensity/Coomassie band intensity}\right)}_{\text{WT}}}$.

Motility is crucial for forming surface-associated multicellular communities by several bacteria, including Salmonella. It helps in the initial surface colonization during biofilm formation (32). As the CRISPR and *cas* deletion mutants showed reduced biofilm formation at 24 h (early time point), we assessed their motility by using a swarming assay. There was at least a 20% reduction in swarming rates of all the knockout strains compared to WT (Fig. S6 and Fig. 3A). The complementation of *ΔcrisprI* and *ΔcrisprII* with corresponding genes restored the defect in their motility (Fig. S6 and Fig. 3A). We next analyzed the expression of flagellin protein (FliC) for the planktonic and pellicle bacteria. The immunoblot analysis revealed that the FliC expression in planktonic bacteria was less for knockout strains than that for the WT. However, in the 96 h pellicle, no FliC expression was observed in all the strains (Fig. 3B).

### (ii) Deletion of CRISPR-Cas genes affects the LPS structure.

The reduction in swarming motility in the knockout strains is not consistent with FliC expression. For example, expression of FliC protein was minimum in the *ΔΔcrisprI crisprII* strain, but its swarming rate was not the lowest. This anomaly could partially be attributed to the variations in the wettability factor, like LPS, that governs the swarming rate. Additionally, the O-antigen of LPS plays a crucial role in biofilm formation (33), and Gram-negative bacteria modify their LPS while in the biofilm (34). Thus, we assayed the LPS profiles of all the knockout strains and compared them with that of the WT (Fig. S7). The intensity of the lipid A band was similar in all the strains except for *Δcas op.* and *ΔΔcrisprI crisprII*. The O-antigen profile showed variations where the ladder-like banding patterns in *ΔcrisprII* and *ΔΔcrisprI crisprII* were less intense than those of the other strains. The band corresponding to very long O-antigen was absent in *ΔcrisprI*, whereas the WT and *Δcas op.* bands had comparable intensities. The very long O-antigen band intensity was similar for *ΔcrisprII* and *ΔΔcrisprI crisprII* but was less than that of WT. As for the banding pattern of core glycoforms, *ΔcrisprII* and WT were similar to *ΔΔcrisprI crisprII* and *Δcas op.*, respectively. *ΔcrisprI* had a distinct pattern of core glycoforms.

All these observations point to alterations of the O-antigen chain in the knockout strains during biofilm formation.

### (iii) The CRISPR-Cas knockout strains show increased pellicle formation due to increased bacterial biomass and its respective components.

**FIG 4:**
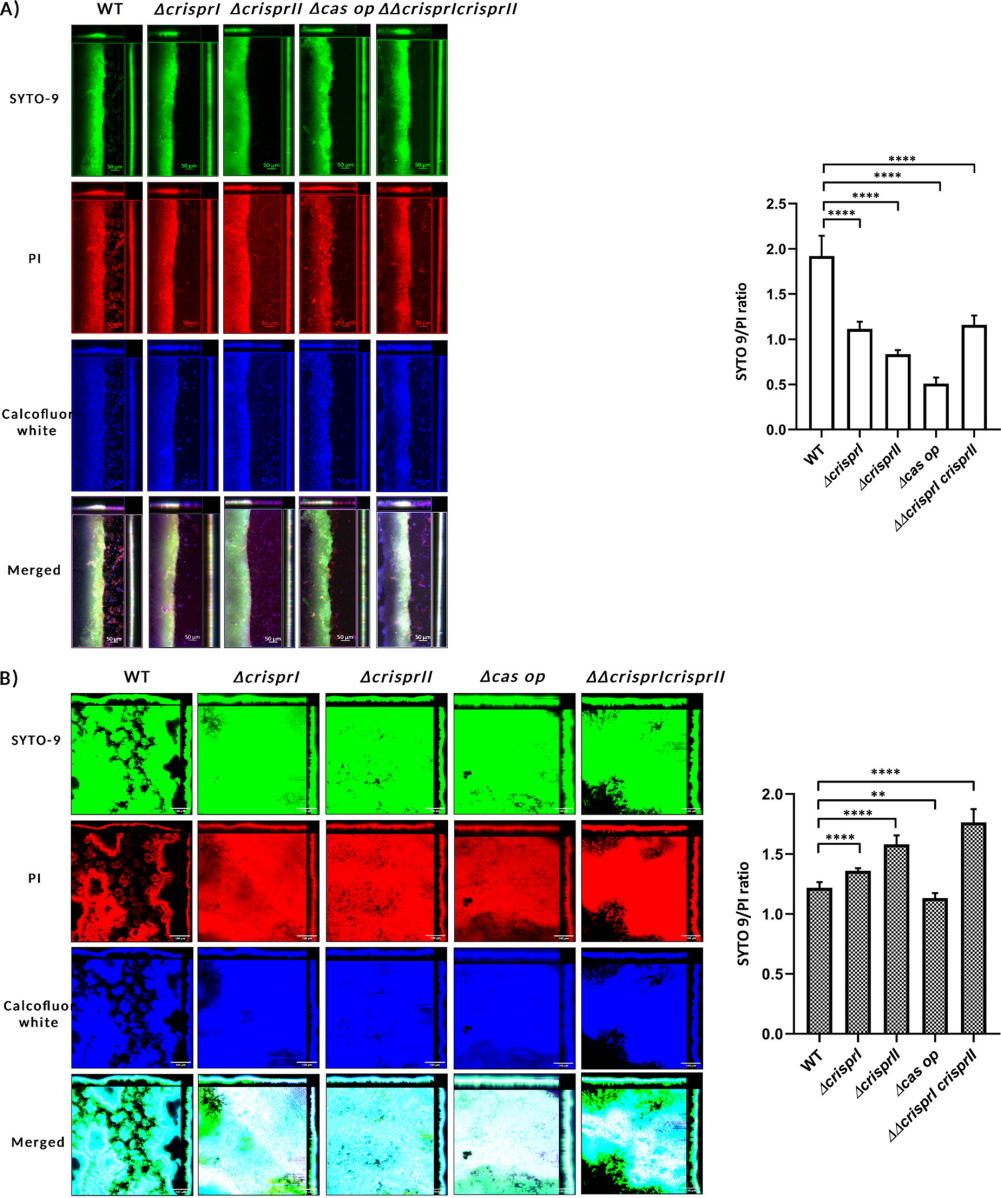
The CRISPR-Cas knockout strains showed temporal variations in their bacterial cell concentration, cellulose content, and SYTO9/PI ratio compared to WT at early (24 h) (A) and late (96 h) time points (B). (A and B) The *S*. Typhimurium strain 14028s wild-type (WT), CRISPR (*ΔcrisprI*, *ΔcrisprII*, and *ΔΔcrisprI crisprII*), and *cas* operon (*Δcas op.*) knockout strains were cultured in LB without NaCl for 24 h (A) and 96 h (B) at 25°C under static conditions. The biofilm formed was stained with SYTO9, propidium iodide (PI), and calcofluor white for 30 min in the dark at RT. The CLSM images were captured, and orthogonal projections of wild-type and CRISPR-Cas knockout strains were obtained. Graphs on the right of the CLSM images represent the ratio of mean intensity of SYTO9 to mean intensity of PI for respective strains at 24 h (A) and 96 h (B). An unpaired *t* test was used to determine significant differences between the WT and knockout strains. Error bars indicate SD. SD. Statistical significance is shown as follows: *, *P* ≤ 0.05; **, *P* ≤ 0.01; ***, *P* ≤ 0.001; ****, *P* < 0.0001; and ns, not significant. A.U., arbitrary units.

The dry weights of the pellicle biofilms by all the knockout strains were similar to those of the WT at 48 h, whereas they were significantly higher at 96 h (Fig. S8A). The temporal variations in the dry weight of all the strains were similar to that of the biofilm formation as estimated using crystal violet assay. As the dry mass comprises bacterial cells and ECM, we independently assessed the bacterial cell mass (by assessing viability) and concentration of the ECM components. The resazurin cell viability assay results show that the knockout strains were more viable than the WT (Fig. S8B), hinting at more bacterial mass. We also validated high bacterial abundance within pellicle biofilm of knockout strains using SYTO9 staining while also assaying the total bacterial abundance within surface-attached biofilm at 24 h (Fig. 4A). The surface-attached biofilm of all the knockout strains (at an early time point, 24 h) had lower SYTO9 intensity than that of the WT (Fig. S8C; Fig. 4A), whereas, at 96 h, SYTO9 intensity was higher than that of the WT (Fig. S8C; Fig. 4B). Further, the SYTO9/propidium iodide (PI) ratio was less for surface-attached biofilm (Fig. 4A) and more for pellicle biofilm (Fig. 4B) of all the knockout strains except *Δcas op.* This indicates that the knockout strains have fewer viable bacteria than the WT in surface-attached biofilm (24 h), while the opposite was true for the pellicle biofilm at 96 h (Fig. 4B and Fig. S9B). The thicknesses of the surface-attached biofilm (Fig. S9A to D) for WT, *ΔcrisprI*, *ΔcrisprII*, *Δcas op.*, and *ΔΔcrisprI crisprII* were 102 μm, 82 μm,62 μm,56 μm, and 68 μm, respectively, whereas, for the pellicle biofilm, the observed thicknesses were 82 μm, 96 μm, 88 μm, 120 μm, and 124 μm, respectively (Fig. S10A to D).

We next estimated the net content of the extracellular polymeric substances like proteins, DNA, and polysaccharides that comprise the ECM. The pellicle biofilms of all the knockout strains had significantly higher polysaccharide concentrations than those of the WT (Fig. S8E). Similarly, the protein concentrations were significantly high in the pellicle biofilms of all the knockout strains except in *ΔΔcrisprI crisprII* (Fig. S8F). The DNA content was significantly higher only in the pellicle biofilm of *ΔcrisprI* and *Δcas op.* (Fig. S8G).

**FIG 5:**
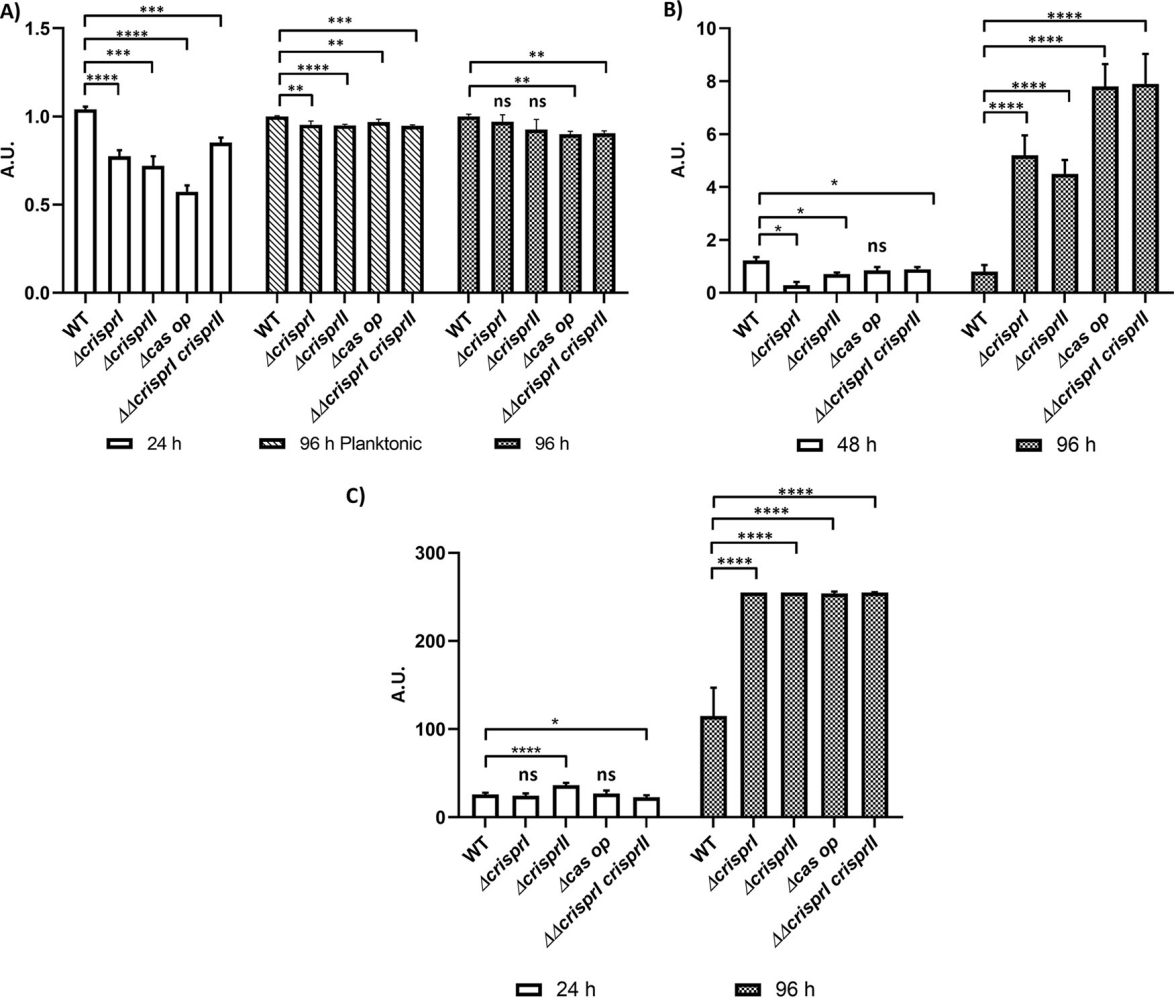
CRISPR-Cas knockout strains showed variations in the production of key components like curli (A) and cellulose (B and C). Curli production in the planktonic culture and pellicle biofilms of wild-type, CRISPR, and *cas* operon knockout strains was assessed with the help of Congo red depletion. The *S*. Typhimurium strain 14028s wild-type (WT), CRISPR (*ΔcrisprI*, *ΔcrisprII*, and *ΔΔcrisprI crisprII*) and *cas* operon *(Δcas op.*) knockout strains were cultured in LB without NaCl for different time periods (24 h and 96 h) at 25°C under static conditions. (A) Congo red depletion was determined by measuring absorbance at 500 nm. Graph represents absorbance at 500 nm of each strain, normalized by absorbance at 500 nm of WT. (B) Cellulose production in the pellicle biofilms of wild-type, CRISPR, and *cas* operon knockout strains was quantitatively assessed by determining the cellulose dry weight in the pellicle biofilm. The *S*. Typhimurium strain 14028s WT, CRISPR (*ΔcrisprI*, *ΔcrisprII*, and *ΔΔcrisprI crisprII*) and *cas* operon (*Δcas op.*) knockout strains were cultured in LB without NaCl for different time periods (48 h and 96 h) at 25°C under static conditions. (C) Qualitative analysis of the amount of cellulose present in the biofilm was done by measuring the calcofluor intensity of the CLSM images (represented in Fig. 4A and B). The *S*. Typhimurium strain 14028s WT, CRISPR (*ΔcrisprI*, *ΔcrisprII*, and *ΔΔcrisprI crisprII*), and *cas* operon (*Δcas op*.) knockout strains were cultured in LB without NaCl 96 h at 25°C under static conditions. An unpaired *t* test was used to determine significant differences between the WT and knockout strains. Error bar indicates SD. Statistical significance is shown as follows: *, *P* ≤ 0.05; **, *P* ≤ 0.01; ***, *P* ≤ 0.001; ****, *P* < 0.0001; and ns, not significant. A.U., arbitrary units.

We further evaluated the expression of individual biofilm components like curli and cellulose. Curli, thin aggregative fimbriae, aid surface adhesion and provide cell-cell interactions while framing the biofilm architecture (35). Less curli production could also be one of the reasons for reduced ring biofilm formation by the knockout strains. Thus, we assessed the curli production (24 h, 48 h, and 96 h) using whole-cell Congo red (CR) depletion assay for planktonic culture and pellicle biofilm. The CR depletion (Fig. 5A and Fig. S11A) was less for both the planktonic culture and pellicle biofilm of all the knockout strains, suggesting low levels of curli protein. The results were further validated using an amyloid-specific indicator dye, Thioflavin-T (ThT) (36). The results confirm that the curli production is less in all the four knockout strains for all the time points tested (Fig. S11B). The cellulose production in surface-attached biofilm was evaluated by quantifying the calcofluor white intensity in the images captured using confocal laser scanning microscopy (CLSM). There was no difference in the cellulose content except for that of *ΔcrisprII* (Fig. 4A, Fig. 5C, and Fig. S9C) for surface-attached biofilm. However, for pellicle biofilm at 96 h, the cellulose content was higher for the knockout strains. We further estimated the cellulose dry weight for the pellicle biofilm at 48 h and 96 h. Interestingly, the cellulose dry weight of the knockout strains was lower than that of the WT at 48 h but was significantly higher at 96 h (Fig. 5B).

The analysis of the orthogonal projections of CLSM images suggests that the cellulose is mainly deposited in the region with mature (multilayered) biofilm containing both the live and dead bacteria. The areas with fresh bacterial growth (less PI staining) have less cellulose. Curli content in the pellicle biofilm is related to surface elasticity, thereby providing mechanical strength to the biofilm (37). As curli protein was less in pellicles of knockout strains, we determined the pellicle biofilm strength using a glass bead assay (supplemental materials and methods) (38). The pellicles of the knockout strains were easily disrupted with less weight while enduring ~50% less weight than the WT pellicles could sustain (Fig. S11C). The results suggest that knockout strains’ pellicles are weaker due to less curli production.

### The CRISPR-Cas knockout strains show altered expression of biofilm-related genes.

To understand the temporal variations in biofilm formation by the CRISPR-Cas knockout strains, we checked the regulation of biofilm-related genes for early (24 h) and late (96 h, pellicle biofilm) time points using RT-PCR.

**FIG 6:**
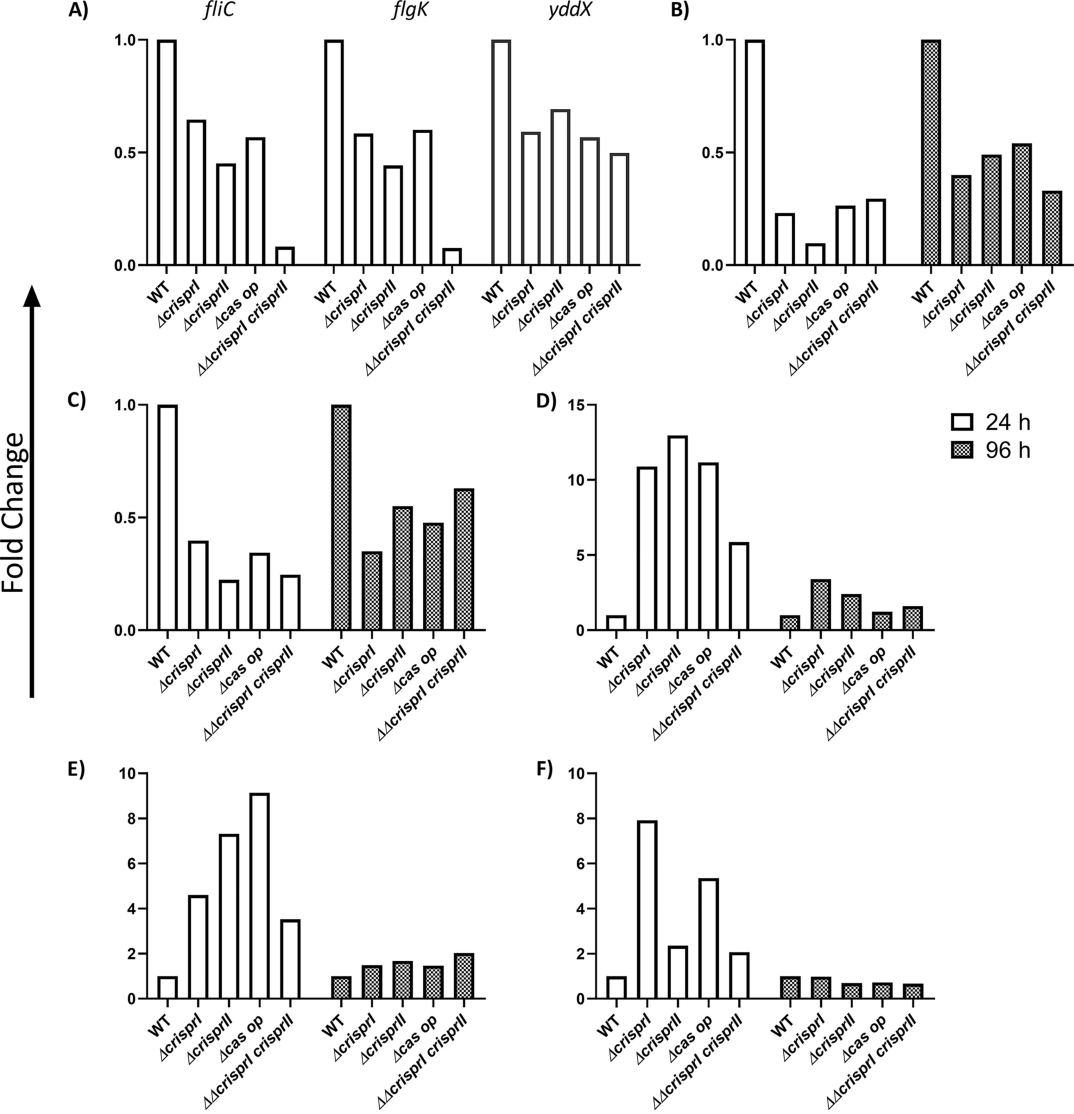
CRISPR-Cas system knockout strains showed differences in the expressions of flagellar genes (A), the production of curli-*csgA* (B) curli-*csgD* (C), LPS-*rfaC* (D), cellulose-*bcsC* (E), and cAMP-regulated protein (*crp*) (F) compared to WT. *S*. Typhimurium strain 14028s wild-type (WT), CRISPR (*ΔcrisprI*, *ΔcrisprII* and *ΔcrisprI ΔcrisprII*), and *cas* operon (*Δcas op.*) knockout strains were cultured in LB without NaCl for different time periods (24 h and 96 h) at 25°C under static conditions. Total RNA was isolated from bacteria (24 h) and pellicle biofilm (96 h). One microgram of RNA was used for cDNA synthesis, followed by qRT-PCR. Relative expression of the gene was calculated using the 2^−ΔΔ^*^CT^* method and normalized to reference gene, *rpoD*.

We first assessed the expression of genes governing motility, such as *fliC* (phase 1 flagellin subunit), *fljB* (phase 2 flagellin) (Fig. S12B), *flgK* (hook protein), *yddX* (biofilm modulation protein, controlling regulatory pathway of flagellar assembly), and *flgJ* (peptidoglycan-hydrolyzing flagellar protein). All the knockout strains showed reduced expression of these genes (Fig. 6A) except *flgJ* (Fig. S12A). Next, to comprehend the observed variations in the LPS profile of the knockout strains (Fig. S7), we analyzed the expression of a few representative LPS genes within the *rfa* (LPS core synthesis) and *rfb* (O-antigen synthesis) gene clusters. *rfaC* (lipopolysaccharide heptosyltransferase I) was upregulated at both the time points (Fig. 6D). However, *rfbG* (DP-glucose 4,6-dehydratase) was upregulated at 24 h (Fig. S12C) and undetected at 96 h, while *rfbI*, coding for the core LPS region, was downregulated at both the time points in all the knockout strains except for *Δcas op.* at 24 h (Fig. S12D).

The *csgA* gene responsible for producing the curli fibers was downregulated at both time points in knockout strains (Fig. 6B). The expression of *csgA* is controlled by the master regulator *csgD*, which, too, had reduced expression in the knockout strains at 24 h and 96 h (Fig. 6C). The expression of the *crp* gene coding for cAMP receptor protein, a *csgD* repressor (39), was high in the knockout strains at 24 h (Fig. 6F) and showed no difference at 96 h. *csgD* also controls the expression of cellulose synthesis genes (*bcsABZC*). Notably, the expression of *bcsA* (cellulose synthase catalytic subunit A) was slightly less (~1.5-fold) in the knockout strains at 24 h (Fig. S12E) and showed no difference at 96 h. Interestingly, *bcsC* (subunit involved in the export of cellulose to the extracellular matrix [40]) was 2-fold upregulated in knockout strains at 24 h (Fig. 6E), and at 96 h, the expression was comparable to the WT. The observed results hint at *csgD*-independent regulation (41) of *bcsC* in the knockout strains.

### The CRISPR-Cas knockout strains export more cellulose in the extracellular milieu.

Since the expression of the cellulose exporter gene, *bcsC*, was high in the knockout strains at 24 h, we estimated the cellulose production, secretion, and incorporation into the pellicle using anthrone assay (Table 2). In accordance with the trend in *bcsA* expression, the total cellulose production was less in the knockout strains at an early time point (24 h), and at the later time points (48 h and 96 h), it became comparable to that of the WT. Intriguingly, at all the time points tested, the intracellular cellulose content was less in the knockout strains than in the WT. In contrast, more cellulose was secreted to the extracellular milieu (culture supernatant). The knockout strains had higher cellulose content in the pellicle biofilm (48 and 96 h) than the WT.

**TABLE 2:** Cellulose production and secretion as estimated by anthrone assay^*a*^

Cellulose content in	Data for strain:				
	WT	*ΔcrisprI*	*ΔcrisprII*	*Δcas op.*	*ΔΔcrisprI crisprII*
At 24 h					
Bacterial pellets	0.210 ± 0.049	0.044 ± 0.004^*g*^	0.064 ± 0.009^*f*^	0.094 ± 0.007^*f*^	0.042 ± 0.010^*g*^
Planktonic supernatant	0.109 ± 0.015	0.075 ± 0.012^*f*^	0.063 ± 0.012^*g*^	0.048 ± 0.013^*g*^	0.067 ± 0.007^*g*^
Pellicle biofilm	NA	NA	NA	NA	NA
Total cellulose^*b*^	0.324 ± 0.057	0.119 ± 0.010^*g*^	0.127 ± 0.004^*g*^	0.143 ± 0.012^*g*^	0.109 ± 0.012^*g*^
% secretion of cellulose^*d*^	35.26	63.40	49.73	33.86	61.43
At 48 h					
Bacterial pellets	0.173 ± 0.012	0.040 ± 0.007^*h*^	0.075 ± 0.007^*h*^	0.090 ± 0.007^*h*^	0.070 ± 0.009^*h*^
Planktonic supernatant	0.539 ± 0.051	0.674 ± 0.030^*g*^	0.584 ± 0.108^*e*^	0.603 ± 0.0701^*e*^	0.59 ± 0.073^*i*^
Pellicle biofilm	0.060 ± 0.004	0.16 ± 0.045^*h*^	0.173 ± 0.059^*h*^	0.129 ± 0.032^*f*^	0.119 ± 0.010^*h*^
Total cellulose^*c*^	0.753 ± 0.084	0.874 ± 0.022^*e*^	0.832 ± 0.055^*i*^	0.822 ± 0.093^*i*^	0.779 ± 0.070^*i*^
% secretion of cellulose^*d*^	77.71	94.43	88.58	86.95	89.32
At 96 h					
Bacterial pellets	0.301 ± 0.038	0.187 ± 0.014^*f*^	0.133 ± 0.044^*f*^	0.224 ± 0.042^*e*^	0.208 ± 0.039^*e*^
Planktonic supernatant	0.141 ± 0.024	0.126 ± 0.031^*i*^	0.168 ± 0.034^*i*^	0.154 ± 0.034^*i*^	0.140 ± 0.042^*i*^
Pellicle biofilm	0.145 ± 0.020	0.267 ± 0.040^*f*^	0.287 ± 0.071^*g*^	0.209 ± 0.011^*f*^	0.218 ± 0.045^*f*^
Total cellulose^*c*^	0.586 ± 0.043	0.580 ± 0.073^*i*^	0.587 ± 0.066^*i*^	0.587 ± 0.033^*i*^	0.565 ± 0.049^*i*^
% secretion of cellulose^*d*^	31.68	40.23	55.82	40.74	40.14

a The given data represents mean ± SD of absorbance at 620 nm. Values represent mean ± SD unless indicated otherwise.

b Total cellulose corresponds to the sum of absorbance for planktonic bacteria (pellet) and culture supernatant.

c Total cellulose corresponds to the sum of recorded absorbance for planktonic bacteria (pellet), culture supernatant, and pellicle biofilm.

d Percentage secretion of cellulose was calculated as $\frac{{\left(\text{planktonic supernatant}\right)}_{\text{strain}}}{{\left(\text{planktonic supernatant}+\text{bacterial pellet}\right)}_{\text{strain}}}\;\times \;100$.

e *P* ≤ 0.05.

f *P* ≤ 0.01.

g *P* ≤ 0.001.

h *P* < 0.0001.

i Not significant.

## DISCUSSION

Biofilm formation in Salmonella is finely regulated, helping the bacteria to sustain various environmental insults while aiding in their persistence within and outside the host (17). Recently, the CRISPR-Cas system has been implicated to play a role in endogenous gene regulation (4)and biofilm formation in various bacteria, including Salmonella (3, 6). Cui et al. demonstrated that Cas3 positively regulates biofilm formation in S. enterica subsp*. enterica* serovar Enteritidis (6). However, our study determined that the Cas proteins negatively regulate biofilm formation in *S*. Typhimurium. This discrepancy in the results could be related to the differences in CRISPR spacers within these serovars (42) or differences in *cas* gene expression observed in both studies. The *cas* genes were upregulated in the *cas3* mutant strain of serovar Enteritidis. This implies that the increased expression of Cas proteins (except Cas3) could have suppressed biofilm in serovar Enteritidis, while in our study, the entire operon was deleted; thereby, there was no *cas* gene expression and, hence, enhanced biofilm formation. Furthermore, our study also demonstrated that CRISPR-I and CRISPR-II arrays negatively regulated pellicle biofilm formation in S. Typhimurium. Correspondingly, a study by Medina et al. suggests that the CRISPR-Cas system suppresses the surface biofilm formation (at 24 h) in *S*. Typhi (7). Intriguingly, we found that the CRISPR-Cas system of *S*. Typhimurium positively regulates surface biofilm while repressing pellicle biofilm. We speculate that the difference in our data on surface biofilm and that of Medina et al. could be because serovars Typhi and Typhimurium differ in arrangement and sequence of *cas* genes, as well as in the CRISPR-I array (6, 7). Could the differential evolution of the CRISPR-Cas system possibly be the reason for the two serovars’ distinct biofilm phenotypes? Or could it be due to differences in the CRISPR spacers? These deductions need further exploration.

We next explored the underlying mechanisms of biofilm regulation by the CRISPR-Cas system. Biofilm formation is a complex mechanism requiring coordination between multiple factors and processes. Flagellar motility is essential for cell-cell adhesion and the formation of microcolonies at the initial stages (32). Our study showed that the CRISPR-Cas knockout strains are less motile, thereby explaining less surface-attached biofilm formation at 24 h by CRISPR-Cas knockout strains. The observations were further validated by the CLSM data of SYTO9 intensity of surface-attached biofilm. Nevertheless, as the biofilm progresses, the requirement of flagella becomes negligible, and its expression is repressed (43). In accordance, we found that FliC expression was absent in pellicle biofilms of all the strains at 96 h. The FliC subunit is also crucial for cholesterol binding and biofilm development on gallstones (44, 45). The decreased biofilm formation by the CRISPR-Cas knockout strains in tube biofilm assay could be attributed to decreased FliC expression. The reduction in FliC expression is also reflected in reduced swarming motility of the knockout strains, but it is not proportionate to the observed trend in FliC expression. For example, despite showing minimal FliC expression among all knockout strains, *ΔΔcrisprI crisprII* had considerable swarming motility. This disparity could be due to variation in the production of LPS and exopolysaccharides. LPS acts as a wettability factor, favoring swarming while inhibiting biofilm formation (46). Interestingly, our study displayed such a relation; all the knockout strains showed reduced swarming but enhanced biofilm formation. Exopolysaccharides, including O-antigen (47) and cellulose (48), function as antidesiccant for the swarmer cells. Together with LPS, these polysaccharides provide a hydration shell around the bacteria, promoting flagellar rotation during swarming (47). Thus, the disparity in the correlation between the FliC expression and swarming motility could possibly be attributed to the differences in the LPS profile and secretion of exopolysaccharides by the knockout and WT strains.

The CRISPR-Cas knockout strains had altered the LPS profile with a difference in the LPS gene expression. The *rfaC* (part of *rfa* gene cluster, responsible for LPS core synthesis) and *rfbG* (part of *rfb* gene cluster, responsible for O-antigen synthesis) genes were upregulated in the knockout strains (24 h). At the same time, *rfbI* was significantly downregulated only in *ΔΔcrisprI crisprII*. Also, studies suggest the plausible conversion of LPS to exopolysaccharides that contribute to external slime (49). The increased exopolysaccharides in the pellicle of CRISPR-Cas knockout strains may also be attributed to this, along with the observed increase in cellulose production. The pellicles formed by the CRISPR-Cas knockout strains are thicker (owing to more bacterial mass and EPS secretion [32]) than the pellicle formed by the WT, confirming the formation of multilayered pellicle biofilms, as evidenced by SEM and CLSM analysis.

As per SEM analysis, the air-exposed pellicle biofilm architecture of *ΔcrisprII* and *ΔΔcrisprI crisprII* appears similar, indicating that *crisprII* could act upstream of *crisprI*. This observation is seconded by our LPS profiling data, where the banding patterns of *ΔcrisprII* and *ΔΔcrisprI crisprII* are similar.

The EPS-overproducing variants reportedly have rough and wrinkled biofilm (50). This supports our observation that the CRISPR-Cas knockout strains overproduce EPS and display intricate wrinkled patterns in the pellicle biofilm. These wrinkled patterns appeared fractal-like (Fig. S13B), as reported for Vibrio cholerae (51). Such morphology could aid bacterial growth of the CRISPR-Cas knockout strains due to the increased surface area that presumably facilitates the nutrient supply (51). Consistently, the bacterial mass was higher in the knockout strains with more viable bacteria, as evidenced by the resazurin assay and SYTO9-PI staining.

The ECM scaffold of pellicle biofilm majorly comprises cellulose and curli that define the long-range and short-range interactions, respectively, thereby providing mechanical integrity (52). The pellicle biofilms of CRISPR-Cas knockout strains have higher cellulose but less curli content. This could probably be the reason for the weaker pellicle biofilm of the CRISPR-Cas knockout strains that quickly collapsed in the glass bead assay. Further, high cellulose in the pellicles of the CRISPR-Cas knockout strains means high water retention that can hamper intermolecular forces in the matrix by decreasing the hydrogen bond interactions. Additionally, less curli could lead to the low tensile strength of the pellicle biofilm of the CRISPR-Cas knockout strains. Higher cellulose and less curli could also explain reduced surface biofilm (ring biofilm at 24 h and 96 h) in the CRISPR-Cas knockout strains. High cellulose may inhibit the formation of surface biofilm, as it can coat the curli fibers required for surface attachment (53). Though the cellulose content was high in pellicle biofilms of the CRISPR-Cas knockout strains, the expression of cellulose synthase, *bcsA*, was 1.5-fold lower in all the knockout strains at 24 h, whereas it was unaltered at 96 h. This corroborates with less cellulose production by the knockout strains at 24 h, which, with time, becomes comparable to that of the WT. However, the intracellular cellulose content in the CRISPR-Cas knockout strains was less than that of the WT, and the percentage secretion of cellulose was higher in the knockout strains for all the tested time points. This could be explained through the upregulated *bcsC* (at 24 h), encoding an exporter of cellulose subunits that could export cellulose units to the extracellular milieu (54). We hypothesized that this secreted cellulose (55, 56) is quickly incorporated into the pellicle, increasing cellulose content in the pellicles of the knockout strains.

The CRISPR-Cas system differentially regulates surface-attached and pellicle biofilm formation via modulation (pink dotted lines) of biofilm-associated genes (*crp*, *yddX*, and *bcsC*). CRP acts on FlhDC, which further governs the expression of class 2 flagellar genes (*flgM* and *fliA*). FlgM inhibits FliA-mediated expression of class 3 flagellar genes. YddX relieves the inhibition of FliA by binding to FlgM, thereby inactivating it. We propose that CRISPR-Cas positively regulates *yddX*, whereby it sequesters FlgM and upregulates the expression of the flagellar subunit. CRP also inhibits CsgD, which, in turn, governs the production of curli and cellulose. Our study suggests that the CRISPR-Cas system mediates the expression of CsgD by suppressing *crp* expression and independently represses the expression of cellulose exporter, BcsC. Taken together, the CRISPR-Cas system enhances flagella and curli production and, hence, surface-attached biofilm formation. Additionally, it suppresses cellulose export to the extracellular milieu, thus negatively regulating pellicle biofilm formation.

Apart from reduced expression of *csgA* and marginal repression of *bcsA*, we found that *csgD*, the activator of *csgBAC* and *bcsABZC*, was also downregulated in the knockout strains. In order to gain mechanistic insight into the CRISPR-Cas-mediated biofilm regulation, we checked the expression of the further upstream regulator, CRP. CRP negatively regulates *csgD* in *S*. Typhimurium (30). The expression of *crp* was significantly upregulated in the knockout strains at an early time point (24 h), explaining the repression of *csgA* and *bcsA*. The conflicting upregulation of *bcsC*, the last gene of *bcsABZC*, could be through the crRNA binding to the *bcsC* gene. The CRISPR spacers (spacers 11, 15, and 19 of the CRISPR-I array and spacers 18 and 26 of the CRISPR-II array) show partial complementarity (43.75% to 65.6%) to the *bcsC* gene (see Fig. S14 in the supplemental material) and, hence, could regulate the expression of *bcsC*. Such a kind of regulation is reported in Pseudomonas aeruginosa, where spacer 12 of the CRISPR-I array has partial complementarity to the *lasR* gene, and the *lasR* gene is regulated by the CRISPR array (57). CRP also activates *flhDC*, a flagellar master operon (58) that further activates the expression of class 2 genes, including *fliA.* The *fliA* gene encodes the flagellar-specific transcription factor σ^28^, which directs the expression of class 3 genes like *fliC* and *flgK*. Before the assembly of the hook-basal body structure, it is held inactive by the anti-σ^28^ factor, *flgM* (59). YddX, a biofilm-dependent modulation protein (BDM) homolog, interacts with FlgM to repress its function as an anti-σ^28^ factor (60). Our study observed a significant downregulation of *yddX* in the knockout strains. Low YddX would mean that FlgM would sequester σ^28^, inhibiting the transcription and expression of class 3 genes, including *fliC* and *flgK.* This explains the impaired motility of the CRISPR-Cas knockout strains.

**FIG 7:**
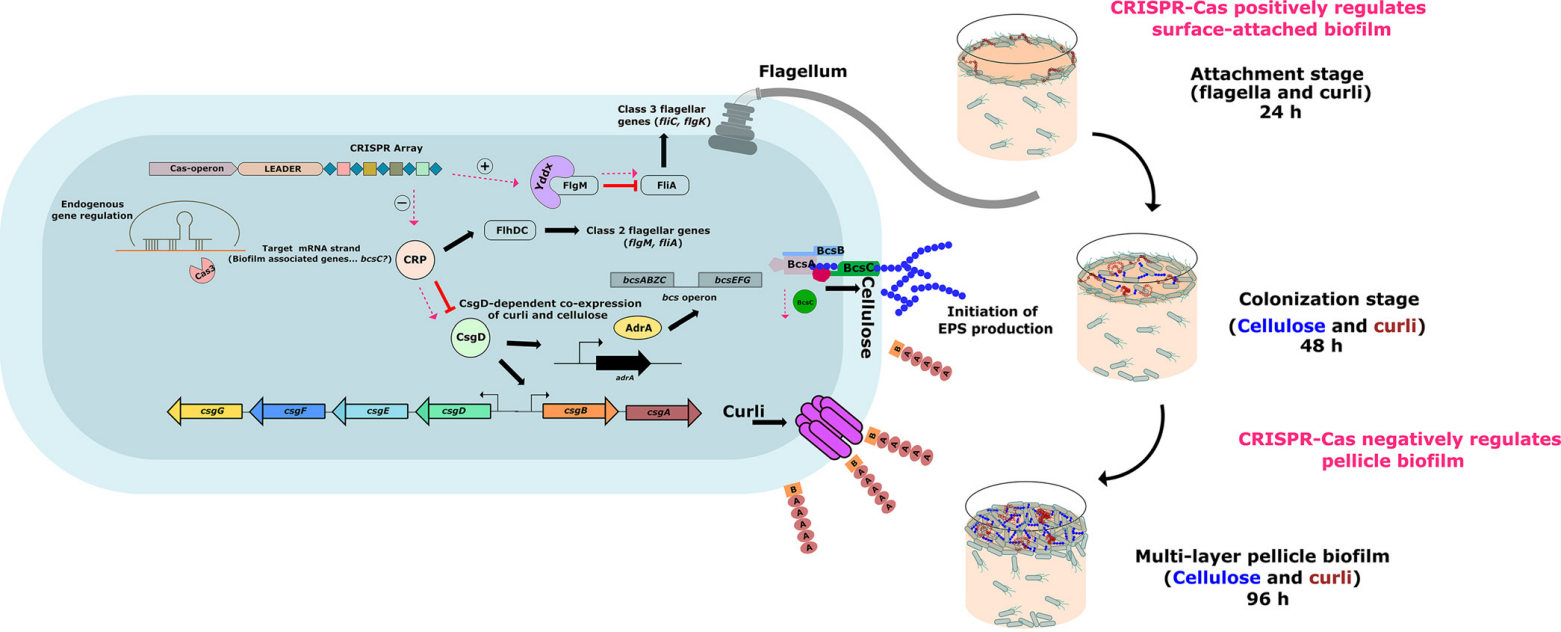
Differential regulation of surface-attached and pellicle biofilm formation in *S*. Typhimurium by the CRISPR-Cas system.

The bacteria transit from the planktonic to biofilm stage under the influence of various regulators that are triggered/repressed by different environmental stresses like those operating within the host and during some stages of food-processing (15). This switch to biofilm formation represents an example of bacterial adaptation to harsh conditions. In Salmonella, CsgD acts like a molecular switch for these modes. It responds to environmental cues like envelop stress (via Rcs), osmolarity (via EnvZ), stationary phase (via RpoS), altered amino acid metabolism (via GcvA/GcvR), glucose availability (via cAMP-CRP), etc. CsgD subsequently activates genes associated with the surface attachment like *csgA* (curli) and represses flagellar genes. In Salmonella, CsgD is repressed by CRP, further repressing the downstream genes like *csgA* and *bcsA*. Our results indicate that under osmolarity stress (LB media without NaCl) generally seen in the food industry, nutrient-deprived conditions (like tryptic soy broth [TSB] media diluted 10 times; Fig. S3), generally seen as hostile host, and bile stress, the CRISPR-Cas system facilitates surface-attached biofilm by suppressing CRP production and the flagellar genes. Further, the system represses the formation of pellicle biofilm by affecting the EPS deposition in the ECM. Hence, the biofilm architecture and nutrient supply to the bacteria within the biofilm. The proposed mechanism of the CRISPR-Cas regulatory pathways is summarized in Fig. 7.

## MATERIALS AND METHODS

### Bacterial strains and culture conditions.

*S*. Typhimurium strain 14028s was used as a parent strain (wild type [WT] 14028s). The wild-type, CRISPR, and *cas* knockout strains (the knockout construction is explained below) and their corresponding complement strains were routinely grown in Luria-Bertani (LB; HiMedia) medium with appropriate antibiotics (see Table S1 in the supplemental material) at 37°C and 120 rpm. The bacterial strains were also subcultured and grown in biofilm medium (LB without NaCl, 1% tryptone, and 0.5% yeast extract) to observe growth patterns up to 12 h.

### Construction of CRISPR and *cas* operon knockout strains.

We generated the CRISPR and *cas* operon knockout strains, *ΔcrisprI* (CRISPR-I array deleted), *ΔcrisprII* (CRISPR-II array deleted), *ΔΔcrisprI crisprII* (CRISPR-I and CRISPR-II arrays deleted), and *Δcas op.* (*cas* operon deleted) using a one-step gene knockout strategy described by Datsenko et al. (61). A phage lambda-derived Red recombination system (supplied on the pKD46 plasmid) was used to replace the desired genes in *S*. Typhimurium 14028s with a chloramphenicol resistance cassette. The double-knockout strain, *ΔΔcrisprI crisprII*, was constructed by replacing the *crisprI* gene with a kanamycin resistance cassette in the *ΔcrisprII* strain. The deletion of the genes was confirmed using PCR amplification of the genes and the inserted antibiotic resistance cassette (Fig. S1 and S2). The primers used for knockout generation and confirmation are listed in Table S2.

### Generation of complement strains for the knockout.

The *crisprI* and *crisprII* genes were amplified using the respective cloning primers listed in Table S1. The amplified products were cloned into BamHI and HindIII sites of pQE60 (a kind gift from Dipshikha Chakravortty, Indian Institute of Science, India). The positive constructs were transformed into the respective knockout strains to obtain corresponding complement strains, *ΔcrisprI* with p*crisprI* and *ΔcrisprII* with p*crisprII*.

### Biofilm quantification using crystal violet assay.

### (i) Tube biofilm assay.

Overnight-grown bacterial cultures were subcultured at 1:100 ratios in LB supplemented with 3% ox bile (HiMedia). These cultures were added in 1.5 mL microcentrifuge tubes coated with 1 mg cholesterol and subsequently incubated at 37°C under static conditions for 96 h. Every day, the medium was replaced with fresh media (LB with 3% ox bile). The biofilms were quantified using a crystal violet (CV) assay.

### (ii) Ring and pellicle biofilm.

Overnight grown bacterial cultures were subcultured at a 1:100 ratio in LB without NaCl in test tubes and incubated at 25°C under static conditions for 24 h, 48 h, and 96 h. The biofilms were quantified using a CV assay.

### (iii) Crystal violet assay.

The biofilms formed were given washes with phosphate-buffered saline (PBS), dried at 56°C for 30 min, and stained with 1% (wt/vol) CV solution for 20 min. After washing with distilled water, biofilms were quantified by solubilizing the biofilm-bound CV with 30% (vol/vol) glacial acetic acid and recording the absorbance of the solution at 570 nm using Multiskan GO (Thermo Scientific, USA).

### Biofilm dry mass and viability assay.

After the designated time period of incubation, the planktonic culture was decanted and replaced with 50 mL of water. The floating biofilm pellicles were carefully collected with the help of a toothpick into microfuge tubes. These collected pellicles were washed and dried in a hot air oven at 56°C, after which their dry weight was measured.

### (i) Resazurin-based viability assay.

The pellicle biofilms were washed twice with distilled water and stained with resazurin (HiMedia) dye (0.337 mg/mL) for 30 min at room temperature (RT). The resazurin fluorescence was measured using a Fluoroskan Ascent (Thermo Scientific, USA) instrument at excitation (λ_ex_) of 550 nm and emission (λ_em_) of 600 nm.

### Biofilm architecture using field emission scanning electron microscopy.

The pellicle biofilms were allowed to form in the glass tube containing an immersed glass slide. The pellicle biofilms fixed with 2.5% glutaraldehyde were dehydrated with increasing ethanol concentrations. The samples were air-dried, sputter coated with gold, and visualized with FEI ApreoS field emission scanning electron microscope (Oxford Instruments, Netherland).

### Confocal laser scanning microscopy for surface-attached and pellicle biofilm.

The surface-attached and pellicle biofilm was stained with 5 μM SYTO9 (Thermo Scientific), 5 μM propidium iodide (PI) (Thermo Scientific), and 50 μM calcofluor white (Sigma-Aldrich) solution for 30 min, in the dark at RT. Slides were imaged with an LSM 880 confocal microscope (Zeiss, Germany) using Z-stack (ZEN 2.3 lite).

### Motility assay.

Five microliters of overnight culture were spot inoculated at the center of swarm petri plates (20 g/L Luria Broth, 0.5% [wt/vol] agar, and 0.5% [wt/vol] glucose). After 45 to 50 min of air drying, the plates were incubated at 37°C for 9 h. The swarm rate was estimated by calculating the radius of the growth front using ImageJ Software (U.S. National Institutes of Health, USA).

### Evaluation of the expression of flagellar proteins.

Planktonic bacterial cells and pellicle biofilms were lysed in Laemmli buffer (50 mM Tris-HCl, pH 6.8, 2% SDS, 10% glycerol, 5% β-mercaptoethanol, and 0.05% bromophenol blue). Pellicle biofilms (96 h) were homogenized with TissueLyser LT (Qiagen, Germany) at 50 kHz for 10 min. An equal amount of each lysate (50 μg protein from planktonic and 400 μg from pellicle biofilm) was processed for immunoblotting using an anti-flagellin antibody (Difco). The immunoblots were developed, and images were captured with the ChemiDoc XRS+ system (Bio-Rad Laboratories, USA). Each immunoblot band was normalized to Coomassie-stained bands, and the relative ratio of each with WT was quantified using Image Lab software (Bio-Rad Laboratories, USA).

### Cellulose determination.

Cellulose dry weight estimation, calcofluor binding, and anthrone assay were used to estimate cellulose content in the planktonic culture and pellicle biofilm. For cellulose dry weight estimation, pellicle biofilms were washed twice with distilled water and hydrolyzed with 0.1 M sodium hydroxide (NaOH) at 80°C for 2 h. The samples were dried and weighed.

### (i) Cellulose quantification by calcofluor.

The pellicle biofilms were rinsed twice with distilled water and stained with a 50 μM calcofluor white stain (Sigma-Aldrich) for 40 min in the dark at RT. The bound calcofluor was measured at λ_ex_ of 350 nm and emission λ_em_ of 475 nm with a Victor3 1420 multilabel counter (PerkinElmer, USA).

### (ii) Cellulose quantification by anthrone.

Planktonic bacteria were pelleted via centrifugation, and the culture supernatant was lyophilized in a lyophilizer (ScanVac freeze dryer). The bacterial pellet, lyophilized supernatant, and pellicle biofilm were resuspended in 300 μL of an acetic-nitric reagent and incubated for 30 min at boiling temperatures. The pellets were then washed twice with sterile water, followed by adding 67% sulfuric acid with intermittent mixings and incubated at RT for 1 h. The samples were placed on an ice bath, and 1 mL of cold anthrone reagent (Fisher Scientific) was added and mixed gently. The tubes were incubated in a boiling water bath for 15 min, after which they were placed on ice. The absorbance at 620 nm was recorded with Multiskan GO.

### Whole-cell Congo red depletion assay.

The planktonic culture and pellicle biofilm after 24 h, 48 h, and 96 h were pelleted at 10,000 × *g* for 5 min and resuspended in Congo red solution (10 μg/mL). After 10 min incubation at RT, the cells were centrifuged at 10,000 × *g* for 10 min. The absorbance of the supernatant was measured at 500 nm with Multiskan GO.

### Curli estimation by Thioflavin-T fluorescence.

The pellicle biofilms were lysed with a lysis buffer (Tris-EDTA, pH 7.5, and 2% SDS) at 95°C for 45 min. The insoluble pellet was washed twice with autoclaved water and resuspended in PBS containing DNase (1 mg/mL; HiMedia) and RNase (20 mg/mL; HiMedia). After 6 h of incubation at RT, the samples were treated with 2 μM ThT (Sigma-Aldrich) for 15 to 20 min in the dark. The absorbance was measured at λ_ex_ of 440 nm and λ_em_ at 482 nm with the Victor3 1420 multilabel counter.

### Quantitative real-time PCR.

Total RNA from 24 h (bacterial culture) and pellicle biofilm in LB without NaCl were isolated using TRIzol reagent (HiMedia) and the method described below, respectively. The RNA extraction was followed by cDNA synthesis using ProtoScript II reverse transcriptase (NEB). Quantitative real-time (qRT-PCR) was performed using PowerUp SYBR Green master mix (Thermo Fisher Scientific). Relative expression of the gene was calculated using the threshold cycle method (2^−ΔΔ^*^CT^*) by normalizing to reference gene *rpoD.* The primers used in RT-qPCR are listed in Table S2.

### (i) RNA isolation from pellicle biofilm.

Pellicle biofilms were resuspended in a solution containing 70% ammonium sulfate and 10% cetyltrimethylammonium bromide (CTAB). The pellicles were crushed with the help of a toothpick and incubated at RT for 10 min. The suspensions were then centrifuged and resuspended in 500 μL of lysis solution (10 mM Tris, 10 mM EDTA, and 1 mg/mL lysozyme) and incubated at RT for 10 min. A mixture of 10% SDS and 3 M sodium acetate was added to the samples. Finally, the RNA was purified using phenol-chloroform-isoamyl alcohol extraction. The RNA in the aqueous phase was then precipitated overnight at −80°C using isopropanol. The purified RNA was then used for cDNA synthesis.

### Statistical analysis.

Statistical analysis was performed using Prism 8 software (GraphPad, California). Unpaired Student's *t* test was performed. Error bars indicate standard deviation (SD). Statistical significance is shown as follows: *, *P* ≤ 0.05; **, *P* ≤ 0.01; ***, *P* ≤ 0.001; ****, *P* < 0.0001; and ns, not significant.
