# Supplementary material for: The CRISPR-Cas System Differentially Regulates Surface-Attached and Pellicle Biofilm in Salmonella enterica Serovar Typhimurium
Source: Microbiol Spectr. 2022 Jun 9;10(3):e00202-22. doi: 10.1128/spectrum.00202-22 (PMC9241790; doi:10.1128/spectrum.00202-22)
Supplement: Supplemental file 1 — Supplemental material. Download spectrum.00202-22-s0001.pdf, PDF file, 5.5 MB [file spectrum.00202-22-s0001.pdf]

Material and Methods

Analyzing Lipopolysaccharide (LPS) profiles

LPS lysis buffer (2 mL of 20% SDS, 800 µL ß-Mercaptoethanol, 200 µL bromophenol, 2 mL glycerol, 15 mL of 1M Tris-HCl) was added to the pellicle biofilms and were rinsed twice with distilled water. The samples were then lysed using Tissue Lyser LT (QIAGEN, Germany) at 50 Hz for 10 mins. The lysates were heated at 100°C, 10 mins followed by DNase (1 µg/µL), RNase (20 µg/µL), and Proteinase-K (20 µg/µL) treatment. Crude LPS [1] thus obtained was resolved using SDS–polyacrylamide gel electrophoresis (SDS-PAGE) with 15% separating gel. The LPS profile was detected using ProteoSilver Silver stain Kit (SIGMA-ALDRICH, USA).

Determining Pellicle Strength

The strains were cultured in LB without NaCl media for 96 h, at 25°C, static condition. The pellicle biofilm strength was determined by addition of glass beads (1 mm, HiMedia) using a tweezer until disruption (collapse of pellicle to the bottom). The weight of glass beads that collapsed the pellicle was recorded [2].

Quantification of extracellular matrix (ECM) components

The 96 h pellicle biofilms were washed with sterile water and sonicated on ice, 15 kHz for 30 secs. The samples were centrifuged and supernatant was used for the analysis. The DNA and protein concentrations in the supernatant of each sample were estimated spectrophotometrically using BioSpectrometer® basic (Eppendorf, Germany). The exopolysaccharides were quantified by the phenol-sulphuric acid method [3] followed by absorbance at 490 nm.

Supplementary Table 1: Strains used in this study

Bacterial Strain	Genotype and Characteristics	Source/ref
Salmonella enterica serovars Typhimurium 14028s	WT 14028s	A kind gift from Prof. Dipshikha Chakravortty, Indian Institute of Science, India
ΔcrisprI	WT 14028s *ΔcrisprI*:: Chl (Chl^r)	This study
ΔcrisprII	WT 14028s *ΔcrisprII*:: Chl (Chl^r)	This study
Δcas op	WT 14028s *Δcas operon* :: Chl (Chl^r)	This study
ΔΔcrisprI crisprII	WT 14028s *ΔcrisprI*:: Kan :*ΔcrisprI*::Chl (Kan^r, Chl^r)	This study
ΔfliC	WT 14028s *ΔfliC*::Kan (Kan^r)	Marathe et al., 2016
ΔcsgD	WT 14028s *ΔcsgD* : : Chl (Chl^r)	A kind gift from Prof. Dipshikha Chakravortty, Indian Institute of Science, India
WT60	WT 14028s transformed with empty pQE60 vector	This study

Δcrispr1 60	Δcrispr1 transformed with empty pQE60 vector	This study
Δcrispr1I 60	Δcrispr1I transformed with empty pQE60 vector	This study
Δcas op 60	Δcas op transformed with empty pQE60 vector	This study
ΔΔcrispr1 crispr1I 60	ΔΔcrispr1 crispr1I transformed with empty pQE60 vector	This study
Δcrispr1 +pcrispr1	Δcrispr1 complemented with functional CRISPR I array cloned in pQE60	This study
Δcrispr1I +pcrispr1I	Δcrispr1I complemented with functional CRISPR II array cloned in pQE60	This study

Supplementary Table 2: Primers used in this study

Sl. No.	Primer Name	Nucleotide Sequence
1	*crispr*1 Knockout (Forward)	5' GAGCTGGCGAAGGCGGAAAAAACGTCCTGATATGCTGGTGGTGTAGGCTGGAG CTGCTTCG 3'
2	*crispr*1 Knockout (Reverse)	5' AAATATATAGTTTTAGTGTGTTCCCCGCGCCAGCGGGGCATATGAATATCCTCCTT A 3'
3	*crispr*1 confirmatory (Forward)	5' CGGATAATGCTGCCGTTGGT 3'
4	*crispr*2 Knockout (Forward)	5' CTGCCATTACTGGTACACAGATTATGATTATGCAACGGCTGTGTAGGCTGGAGCT GCTTCG 3'
5	*crispr*2 Knockout (Reverse)	5' GCCTGCCGATGCCGTCTGTGACTCATCCATTACCTTGC CATATGAATATCCTCCTTA 3'
6	*crispr*2 confirmatory (Forward)	5' GCAATACCCTGATCCTTAACGC 3'
7	*cas op*. Knockout (Forward)	5' AGGCGTAGAGTGCTTTTATTATCCACATGCTGGAGTTTACGTGTAGGCTGGAGCT GCTTCG 3'
8	*cas op*. Knockout (Reverse)	5' CAACAGGAAGAAAAGAAACCAAACGCAGTCCATCCAAATC CATATGAATATCCTCCTTA 3'
9	*cas op*. confirmatory (Forward)	5' CTTTGAGCGCTTCTTCCAG 3'

10	Confirmatory Internal Primer	5' CCTCCTTAGTTCCTATTCCG 3'
11	*fliC* (Forward)	5' GATAAGACGAACGGTGAGG 3'
12	*fliC* (Reverse)	5' AGCCTCTGTCAAATCAGC 3'
13	*flgK* (Forward)	5' GGATAACACCACCTTCACG 3'
14	*flgK* (Reverse)	5' CAATCTCGGCTTCATTTGTC 3'
15	*csgA* (Forward)	5' GGATTCCACGTTGAGCATT 3'
16	*csgA* (Reverse)	5' TACTGTTATCCGCACCCT 3'
17	*csgD* (Forward)	5' AACTGGCCTCATATTAACGG 3'
18	*csgD* (Reverse)	5' GTGCGTAATCAGGTAACTGG 3'
19	*bcsA* (Forward)	5' GATGGACATTTGTTCTCCTG 3'
20	*bcsA* (Reverse)	5' GCGTTGGAAAGACATATTCC 3'
21	*bcsC* (Forward)	5' GACCAGTTGAGCGGTAAA 3'
22	*bcsC* (Reverse)	5' GTCGTAATGCCAGATCATGT 3'
23	*rpoD* (Forward)	5' GATAAGACGAACGGTGAGG 3'
24	*rpoD* (Reverse)	5' AGCCTCTGTCAAATCAGC 3'
25	*rfaC* (Forward)	5' TACGATAAACCGCAGTCG 3'
26	*rfaC* (Reverse)	5' CTTCCGGCCAGTGTTTA 3'
27	*rfbG* (Forward)	5' CTTGATGCGCCAACTGTTC 3'
28	*rfbG* (Reverse)	5' AAAGGCTGGGCTGCCATA 3'
29	*yddX* (Forward)	5' AAATACCTCAGCAGCACAACC 3'
30	*yddX* (Reverse)	5' TCTTCAGTGACAACGCCTAAC 3'
31	*crp* (Forward)	5' GGTTCTTGTCTCATTGCCA 3'

32	*crp* (Reverse)	5' CGGAGCCTTTAACGATGTAG 3'
33	*flgJ* (Forward)	5' CGCAATCTCTGAACGAACTG 3'
34	*flgJ* (Reverse)	5' CGCATACTTTTCAGCATCATC 3'
35	*rfbI* (Forward)	5' TATCGGGCTGGTATCCATCTTGA 3'
36	*rfbI* (Reverse)	5' CTTTGGAGTCAACAACTTCTCC 3'
37	*fljB* (Forward)	5' GAGCGTCTGTCTTCTGGT 3'
38	*fljB* (Reverse)	5' TTACGGGAAGCCTGAGTC 3'
39	16s rRNA (Forward)	5' CCTGGACAAAGACTGACGCT 3'
40	16s rRNA(Reverse)	5' TTTAACCTTGCGGCCGTACT 3'
41	*crispr1* expression (Forward)	5' GATAAACCGTGAGCAACGACAG 3'
42	*crispr1* expression (Reverse)	5' GCCCTGCAACGGTTTATCC 3'
43	*crispr2* expression (Forward)	5' GCGTTTGACATGAGCGTGTT 3'
44	*crispr2* expression (Reverse)	5' GGTATAGACCAGCGTCACGG 3'
45	*cas3* expression (Forward)	5' AACATGCCGGTTGGATTTGC 3'
46	*cas3* expression (Reverse)	5' CCACAGCGTGACAGACTCTT 3'
47	*cse2* expression (Forward)	5' TGATGCCTGTTTGGCTGAGG 3'
48	*cse2* expression (Reverse)	5' TGTCGCCACCTTTCTTCTGT 3'

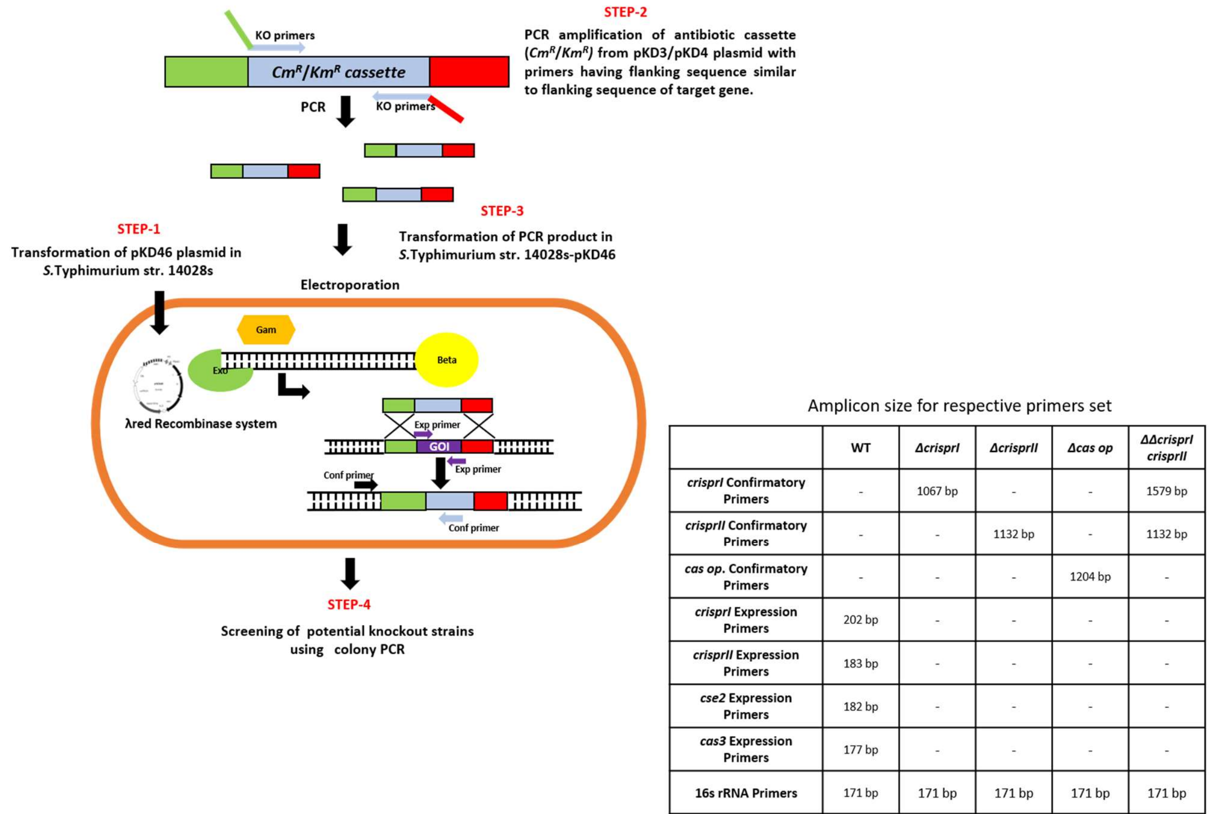

Amplicon size for respective primers set

	WT	*ΔcrisprI*	*ΔcrisprII*	*Δcas op*	*ΔΔcrisprI crisprII*
crisprI Confirmatory Primers	-	1067 bp	-	-	1579 bp
crisprII Confirmatory Primers	-	-	1132 bp	-	1132 bp
cas op. Confirmatory Primers	-	-	-	1204 bp	-
crisprI Expression Primers	202 bp	-	-	-	-
crisprII Expression Primers	183 bp	-	-	-	-
cse2 Expression Primers	182 bp	-	-	-	-
cas3 Expression Primers	177 bp	-	-	-	-
16s rRNA Primers	171 bp	171 bp	171 bp	171 bp	171 bp

Supplementary Figure S1: Schematic representation for generating and confirming the knockout strains. The successful generation of knockout strains (*ΔcrisprI, ΔcrisprII , Δcas op,* and *ΔΔcrisprI crisprII*) would require homologous recombination between the gene of interest (GOI) and the antibiotic resistance cassette. For *ΔcrisprI, ΔcrisprII ,* and *Δcas op* the genes were replaced with chloramphenicol resistance cassette, whereas for generation of *ΔΔcrisprI crisprII,* the *crisprI* gene was replaced with kanamycin resistance cassette in the *ΔcrisprII* strain.

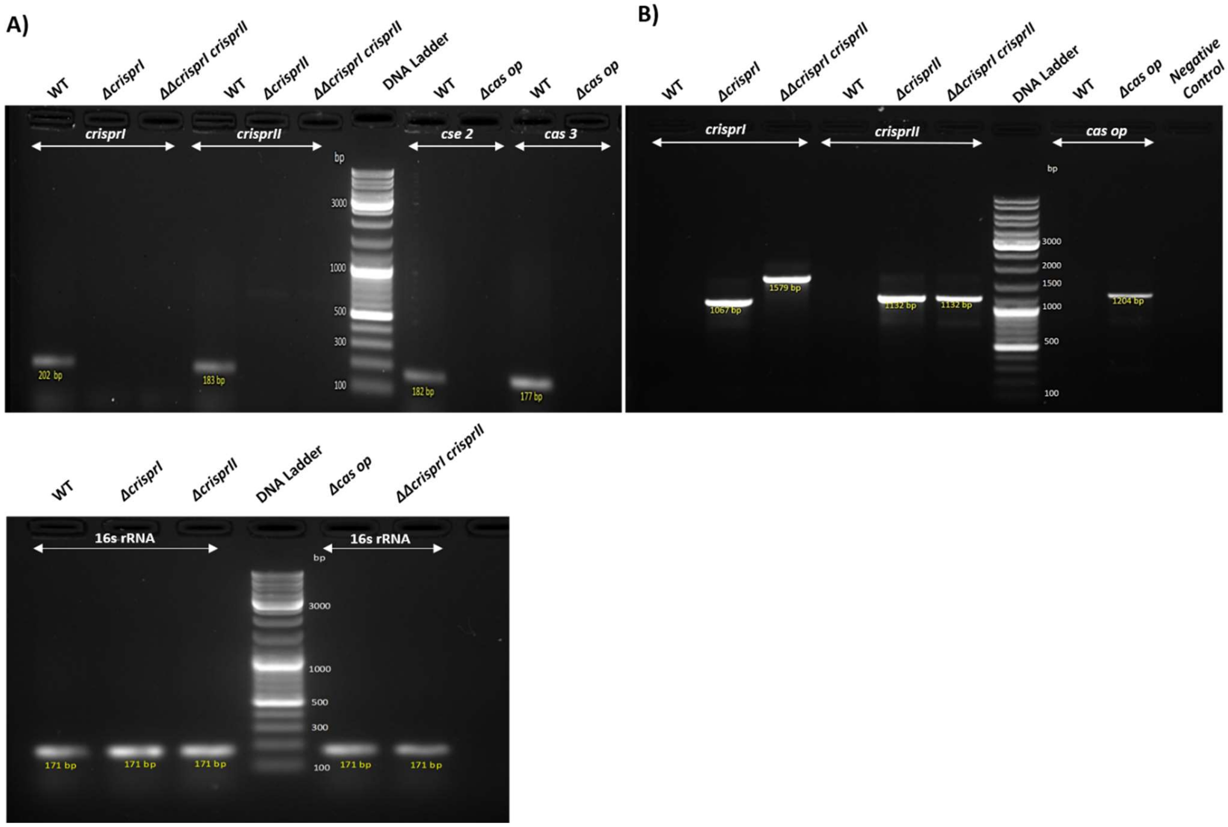

Supplementary Figure S2: The deletion of the CRISPR-Cas components was confirmed through PCR using expression primers (A), and confirmatory primers (B). The colony PCR of potential knockout strains was done using respective primers mentioned in Supplementary Figure S1, and the amplicons were visualized using agarose electrophoresis. **A.** The presence of CRISPR-Cas genes was checked in WT and knockout strains (*ΔcrisprI, ΔcrisprII , Δcas op,* and *ΔΔcrisprI crisprII*), while 16s rRNA was used as a positive control for each strain. **B.** Amplicons of appropriate sizes were obtained for each knockout strain, whereas WT did not yield any bands.

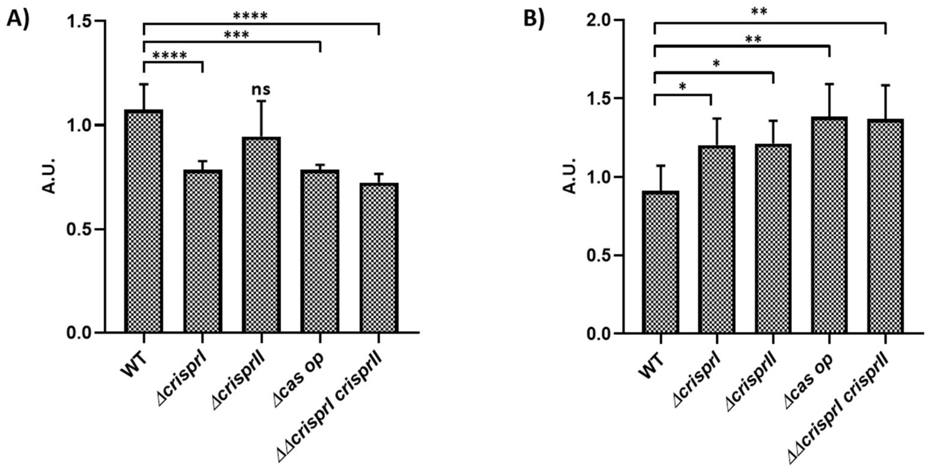

Supplementary Figure S3: The CRISPR-Cas system knockout strains of *S. enterica* subsp. *enterica* serovar Typhimurium 14028s showed reduced biofilm formation at the solid-liquid interface(A), while these strains showed increased biofilm (pellicle) at solid-liquid and air interface (B). The *S.* Typhimurium strain 14028s wild-type (WT), CRISPR (*ΔcrisprI, ΔcrisprII* and *ΔΔcrisprI crisprII*) and *cas operon (Δcas op*) knockout strains were cultured in Tryptic Soy Broth (TSB) media for 96 h, at 25°C, static condition in 24-well plastic plate. The biofilm formation was estimated using crystal violet staining method. The graph represents OD_{570nm} for each strain, normalized by OD_{570nm} of WT. Unpaired t-test was used to determine significant differences between the WT and knockout strains. Error

bars indicate SD. Statistical significance: *≤ 0.05, **≤ 0.01, ***≤ 0.001, ****<0.0001, ns = not significant. A.U., arbitrary units.

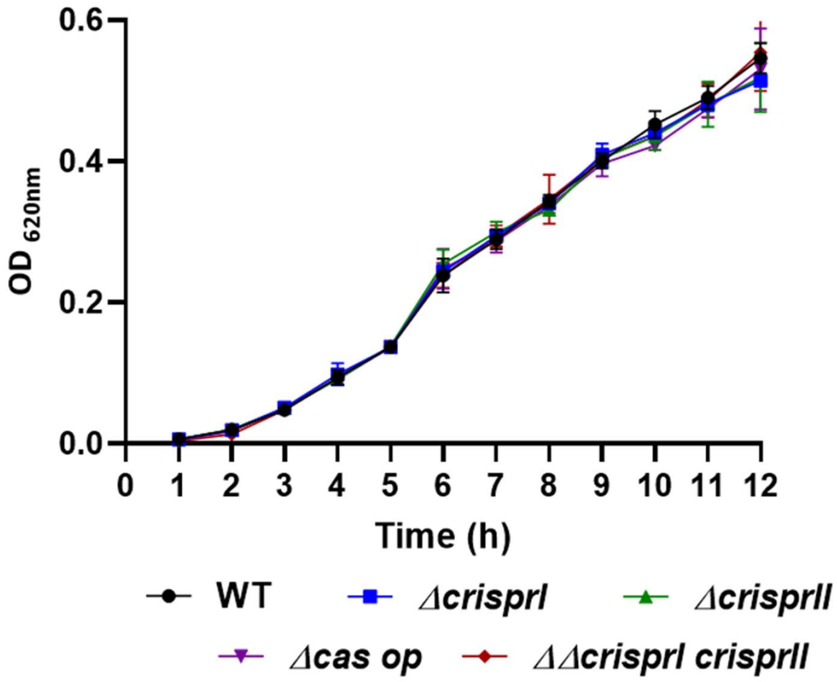

Supplementary Figure S4: The CRISPR- Cas system knockout strains of *S. enterica* subsp. *enterica* serovar Typhimurium 14028s showed a similar growth trend to wild-type in LB without NaCl media. The *S.* Typhimurium strain 14028s wild-type (WT), CRISPR (*ΔcrisprI, ΔcrisprII* and *ΔΔcrisprI crisprII*) and *cas operon (Δcas op*) knockout strains were cultured in LB without NaCl media for 12 h, at 37°C, shaking condition. The graph represents OD_{620nm} for each strain.

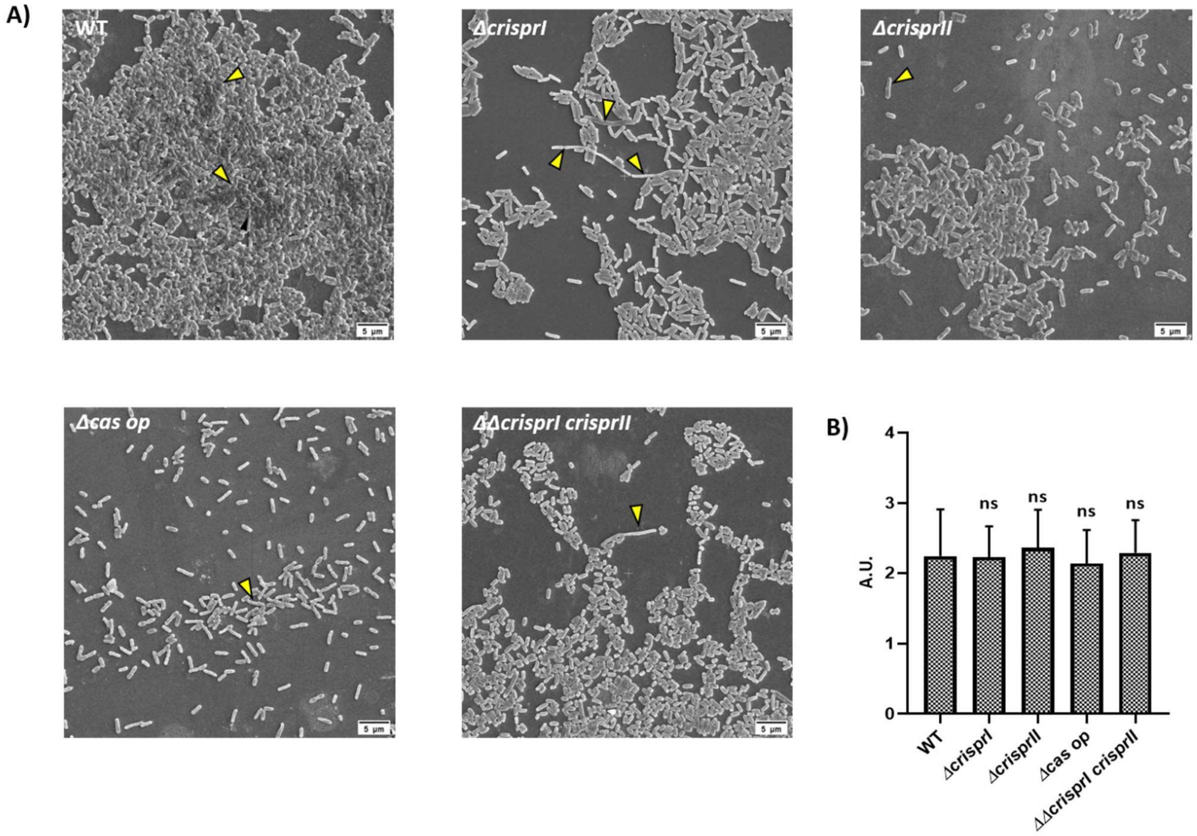

Supplementary Figure S5: Morphology of air-exposed side of surface-attached (glass) biofilm at early (24 h) time point. A. The knockout (*ΔcrisprI, ΔcrisprII, Δcas op,* and *ΔΔcrisprI crisprII*) strains formed patchy bacterial aggregates, in comparison to wild-type (WT), which had tightly packed bacterial aggregates covering larger area, with a few dome-like structure (arrow-head in the WT micrograph). Few elongated cells (arrow-head in the micrographs) were also observed in the biofilms of the knockout strains. The strains were grown in LB without NaCl media for 24 h, at 25°C, static conditions. The pellicle biofilms formed was fixed using 2.5% glutaraldehyde were dehydrated with increasing concentrations of ethanol. The images were captured at 5000x magnification and scaled to bar. **B.** The graph represents the average size (in µm) of WT and Knockout strains. Unpaired t-test was used to determine significant differences between the WT and knockout strains. Error bars indicate SD. Statistical significance: *≤ 0.05, **≤ 0.01, ***≤ 0.001, ****<0.0001, ns = not significant. A.U., arbitrary units.

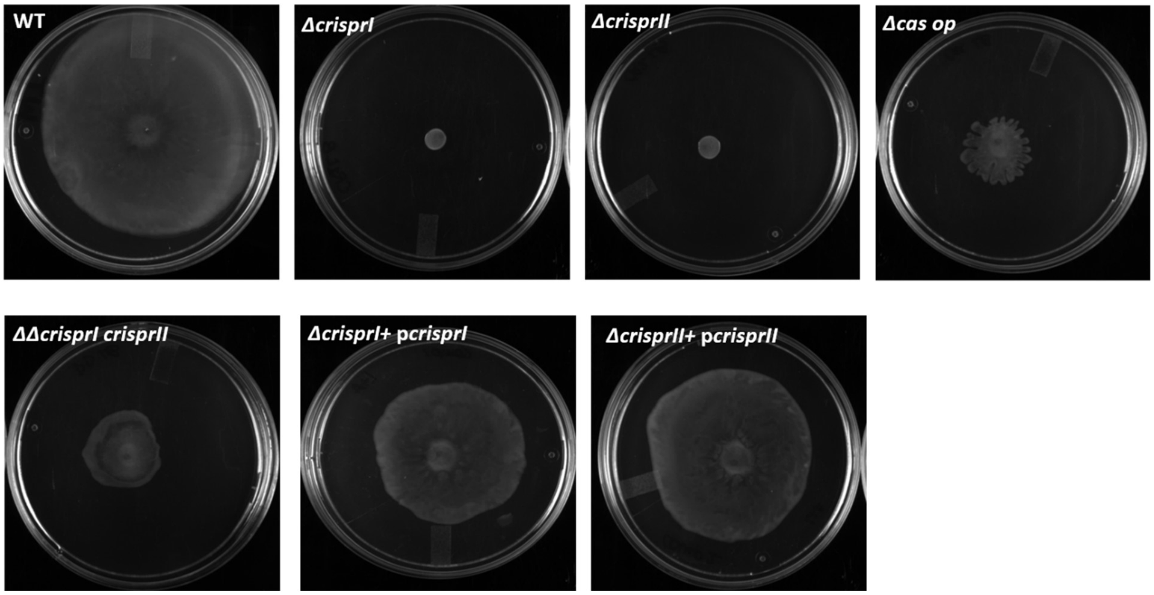

Supplementary Figure S6: CRISPR-Cas system knockout strains show reduced swarming motility. Swarm plates (0.5% agar, 20g/L of LB and 0.5% glucose) were point inoculated with overnight cultures and incubated at 37°C for 9 h. The complement strains (*ΔcrisprI* + p*crisprI, ΔcrisprII*+ p*crisprII*) showed reversal of swarming ability confirming the mutation process was not polar.

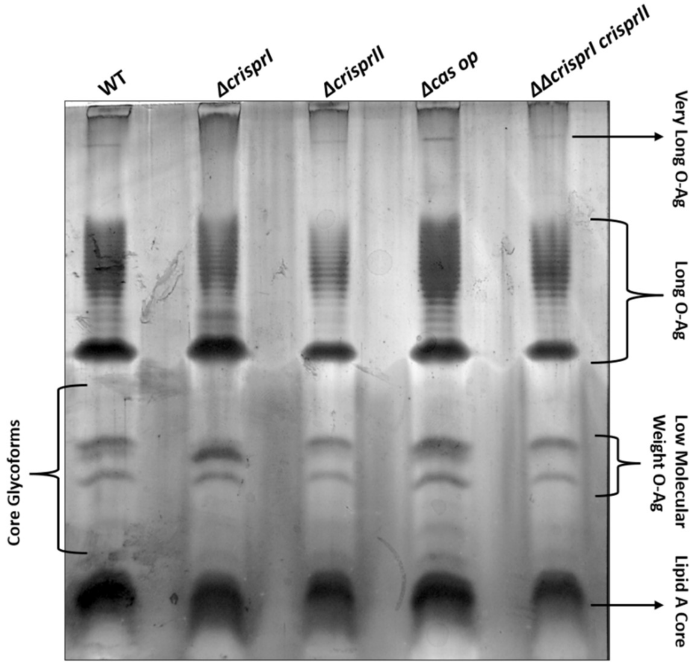

Ratio[#]	WT	*ΔcrisprI*	*ΔcrisprII*	*Δcas op*	*ΔΔcrisprI crisprII*
Very Long O-Ag	1.00	-	0.66	1.08	0.68
Long O-Ag	1.00	1.06	0.55	0.86	0.67
Low Molecular Weight O-Ag	1.00	1.24	0.93	1.03	0.87
Lipid A Core	1.00	0.92	0.88	0.83	0.80

Supplementary Figure S7: Silver-stained Lipopolysaccharide (LPS) profiling of wild-type (WT), and CRISPR-Cas system knockout strains. The variation in O-antigen was analyzed by LPS profiling. The strains were grown in LB without NaCl media for 96 h, at 25°C, static conditions. pellicle biofilm was homogenized, and heated, followed by DNase, RNases and Proteinase-K treatment to extract crude LPS. The processed samples were loaded on 15% SDS-PAGE MIDI gel, which was later stained using a silver staining kit. Variations in banding pattern and intensity between knockout (*ΔcrisprI, ΔcrisprII, Δcas op,* and *ΔΔcrisprI crisprII*) strains and WT were observed in long O-Ag, low molecular weight O-Ag and core glycoforms regions. #Ratio indicates the intensity of the bands observed on the gel for all strains normalized by the intensity of the band for wildtype.

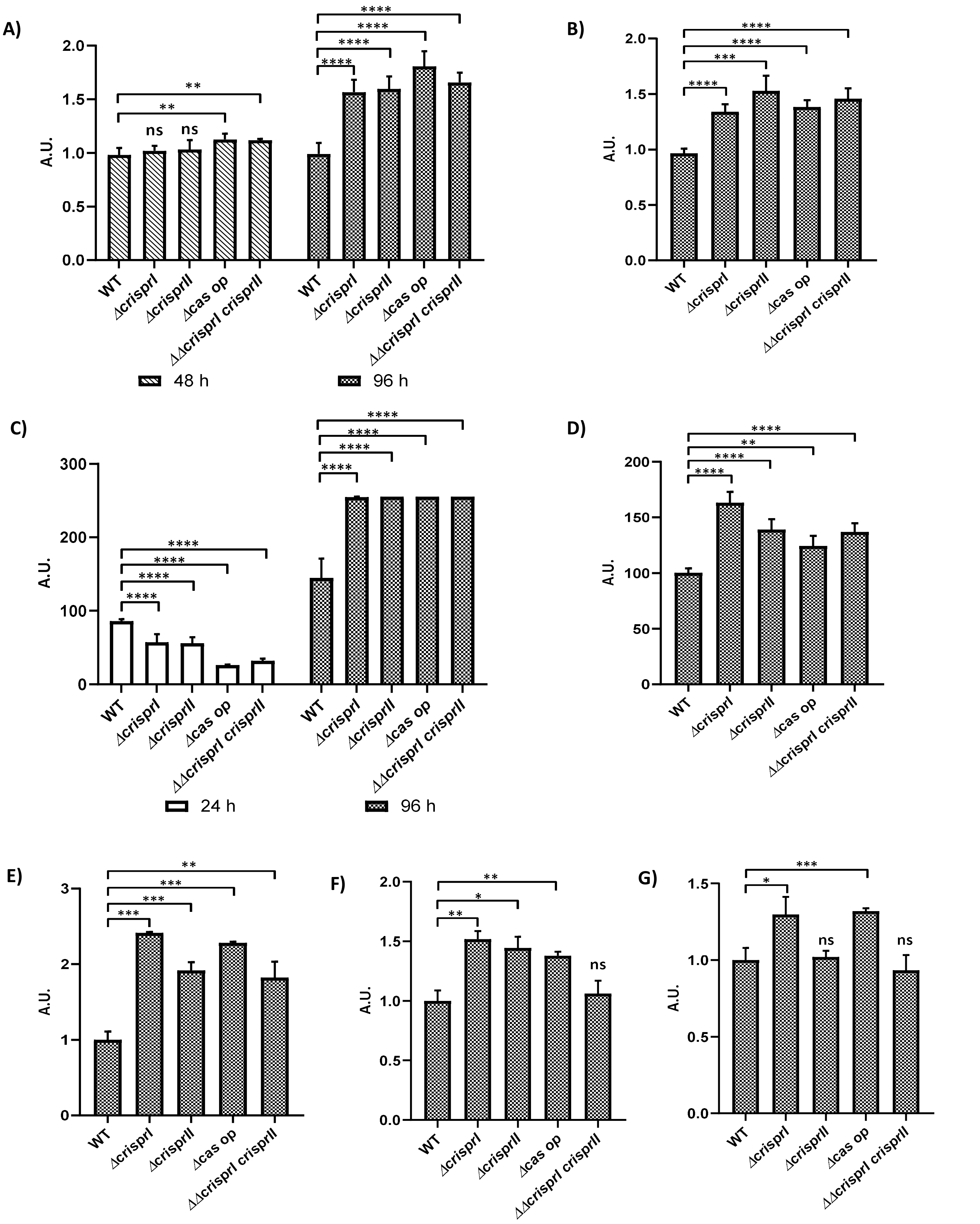

Supplementary Figure S8: Compared to WT, CRISPR-Cas system knockout strains show differences in their bacterial biomass (A), metabolic activity (B), bacterial cell concentration (C), cellulose content in pellicle biofilm (D), and ECM components like polysaccharides (E), protein (F), and DNA (G). A. The *S.* Typhimurium strain 14028s wild-type (WT), CRISPR (*ΔcrisprI, ΔcrisprII* and *ΔΔcrisprI crisprII*) and *cas operon (Δcas op*) knockout strains were cultured in LB without NaCl media for different time periods (48 h, and 96 h) at 25°C, static condition. The biomass of

the strains was estimated with the help of dry weight of pellicle biofilms harvested post 48 h and 96 h incubations. The graph represents dry pellicle biofilm weight (in gms) of each strain normalized by the dry pellicle biofilm weight (in gms) of WT at respective time points. **B.** The metabolic activity was assessed by resazurin assay. *S.* Typhimurium strain 14028s wild-type (WT), CRISPR (*ΔcrisprI, ΔcrisprII* and *ΔΔcrisprI crisprII*) and *cas operon (Δcas op*) knockout strains were cultured in LB without NaCl media for 96 h, at 25°C, static condition. The pellicle biofilm formed after 96 h incubation was stained with resazurin dye and fluorescence was measured using a fluorimeter at excitation (λ_{Ex}) 550 nm and emission (λ_{Em}) of 600 nm. The graph represents fluorescence intensity observed for each strain normalized by fluorescence intensity of WT. **C.** The *S.* Typhimurium strain 14028s wild-type (WT), CRISPR (*ΔcrisprI, ΔcrisprII* and *ΔΔcrisprI crisprII*) and *cas operon (Δcas op*) knockout strains were cultured in LB without NaCl media for different time point (24 h and 96 h), at 25°C, static condition. The pellicle biofilm formed was stained with SYTO 9, for 30 mins in the dark, at RT. The graph represents Mean intensity of SYTO9 observed for each strain. **D.** Qualitative analysis of the amount of cellulose present in the pellicle biofilm was done by measuring the calcofluor bound, at excitation of 350 nm and emission 475 nm. The *S.* Typhimurium strain 14028s wild-type (WT), CRISPR (*ΔcrisprI, ΔcrisprII* and *ΔΔcrisprI crisprII*) and *cas operon (Δcas op*) knockout strains were cultured in LB without NaCl media 96 h, at 25°C, static condition. **E-G.** *S.* Typhimurium strain 14028s wild-type (WT), CRISPR (*ΔcrisprI, ΔcrisprII* and *ΔΔcrisprI crisprII*) and *cas operon (Δcas op*) knockout strains were cultured in LB without NaCl media for 96 h, at 25°C, static condition. **E.** The exopolysaccharides were quantified by the phenol-sulfuric acid method, by measuring absorbance at 490nm. The graph represents absorbance observed at 490nm for each strain normalized by absorbance observed at 490nm for WT. **F & G.** The protein and DNA concentrations in the supernatants of each sample was estimated spectrophotometrically and was further normalized by absorbance for WT in each case. Unpaired t-test was used to determine significant differences between the WT and knockout strains. Error bars indicate SD. Statistical significance: *≤ 0.05, **≤ 0.01, ***≤ 0.001, ****<0.0001, ns = not significant. A.U., arbitrary units.

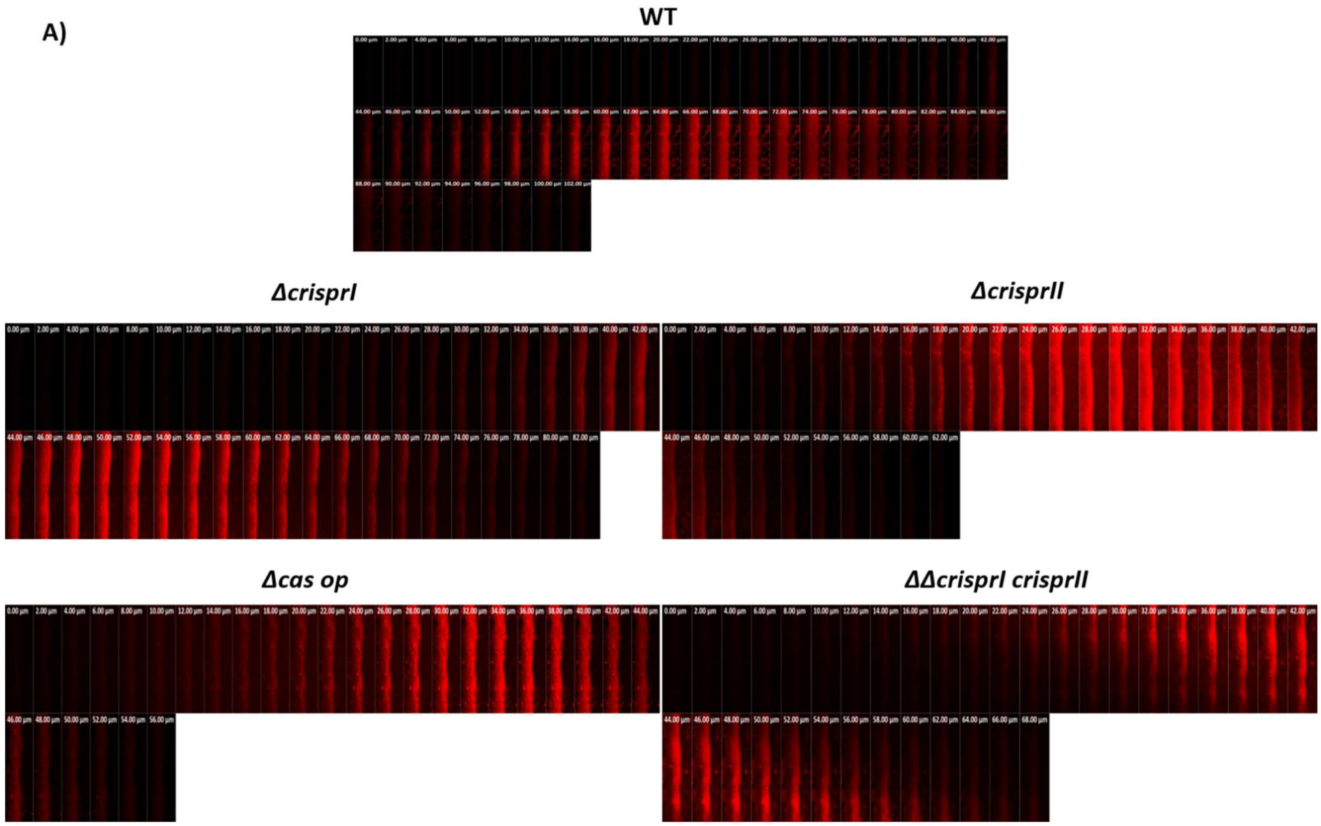

B)

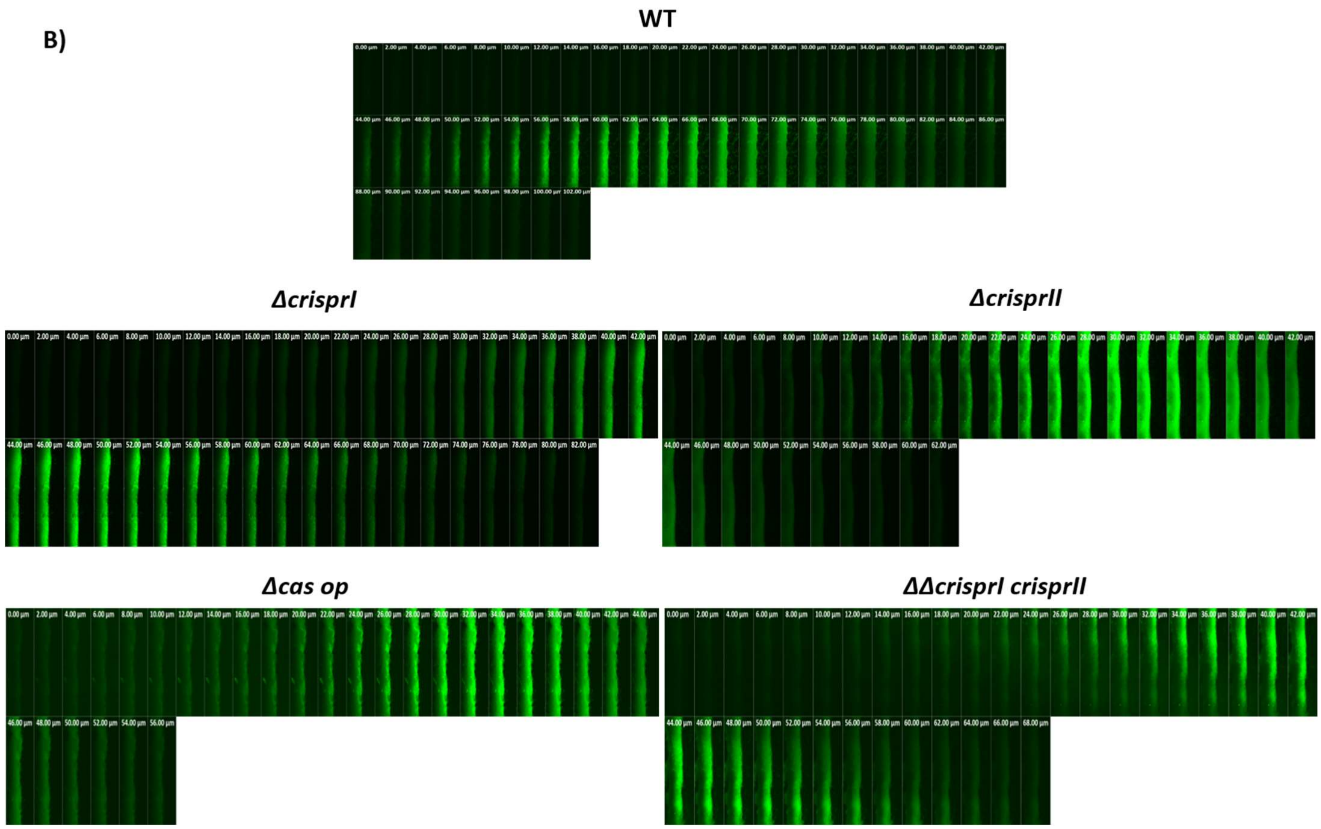
WT
Δcrispr I
Δcrispr II
Δcas op
ΔΔcrispr I crispr II

C)

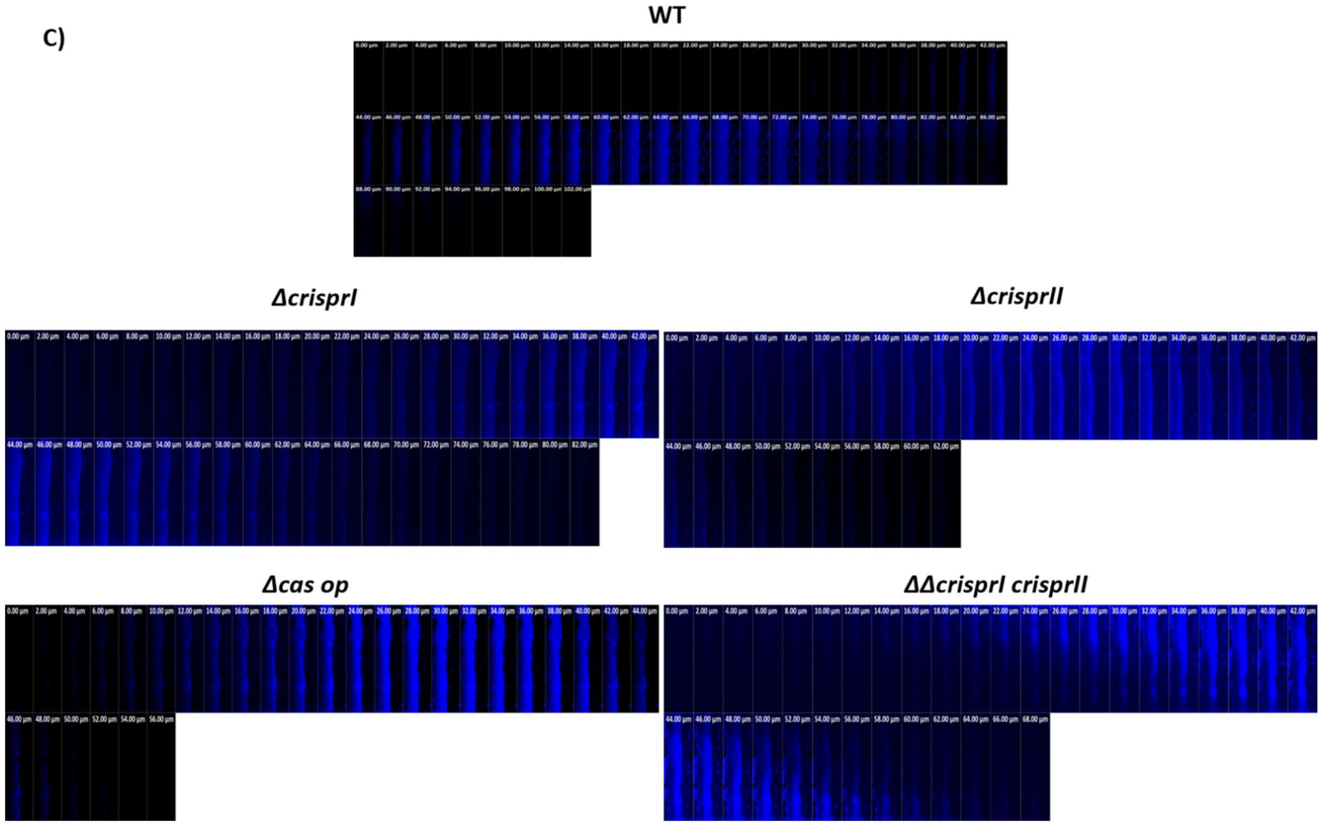
WT
Δcrispr I
Δcrispr II
Δcas op
ΔΔcrispr I crispr II

D)

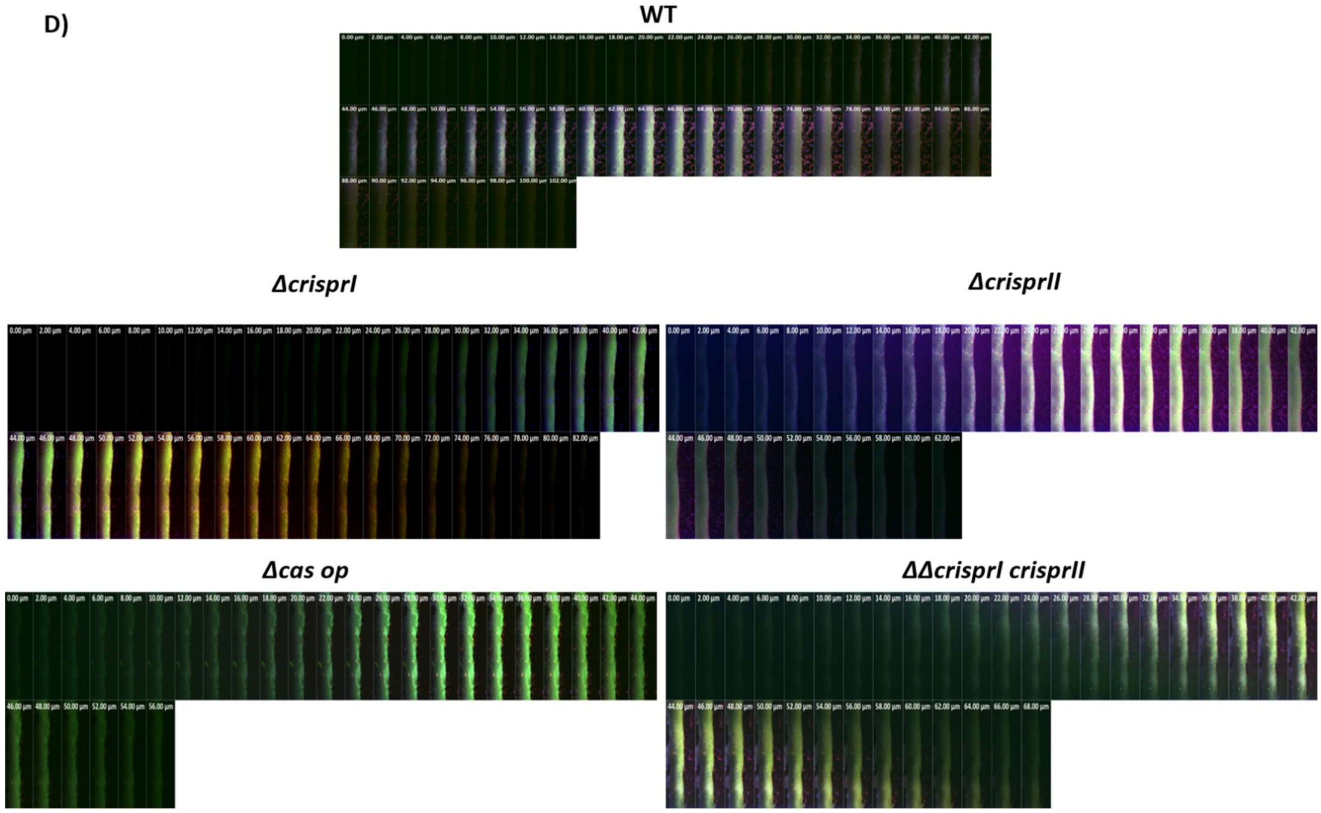

Supplementary Figure S9A-D. CLSM images (stacks) of wild-type and CRISPR- Cas knockout strains stained with Propidium Iodide(A), SYTO 9 (B), Calcofluor white (C). The final panel represents the merged CLSM stacks for all three components (D) The *S.* Typhimurium strain 14028s wild-type (WT), CRISPR (*ΔcrisprI, ΔcrisprII* and *ΔΔcrisprI crisprII*) and *cas operon (Δcas op*) knockout strains were cultured in LB without NaCl media for 24 h, at 25°C, static condition. The pellicle biofilm formed was stained with Propidium Iodide (PI), SYTO 9, and Calcofluor white for 30 mins in the dark, at RT.

A)

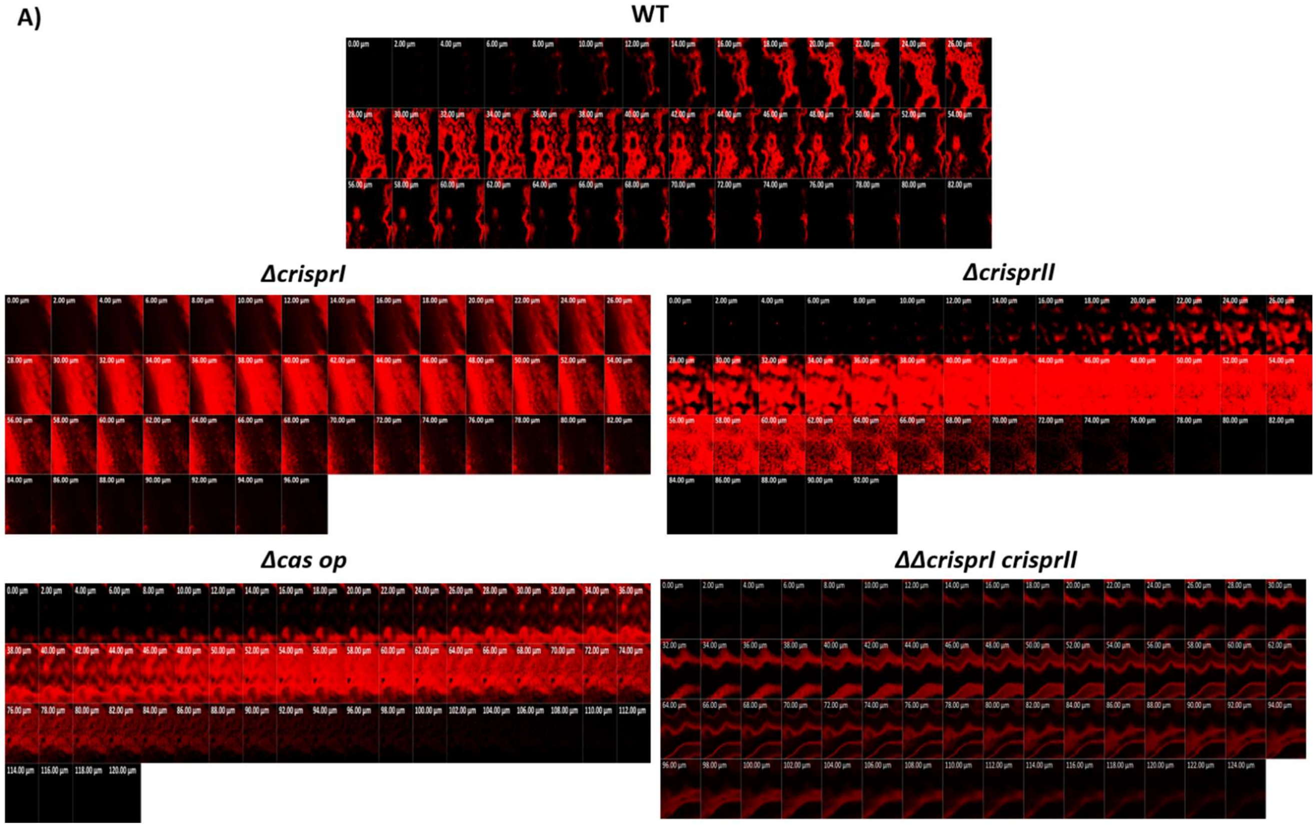

B)

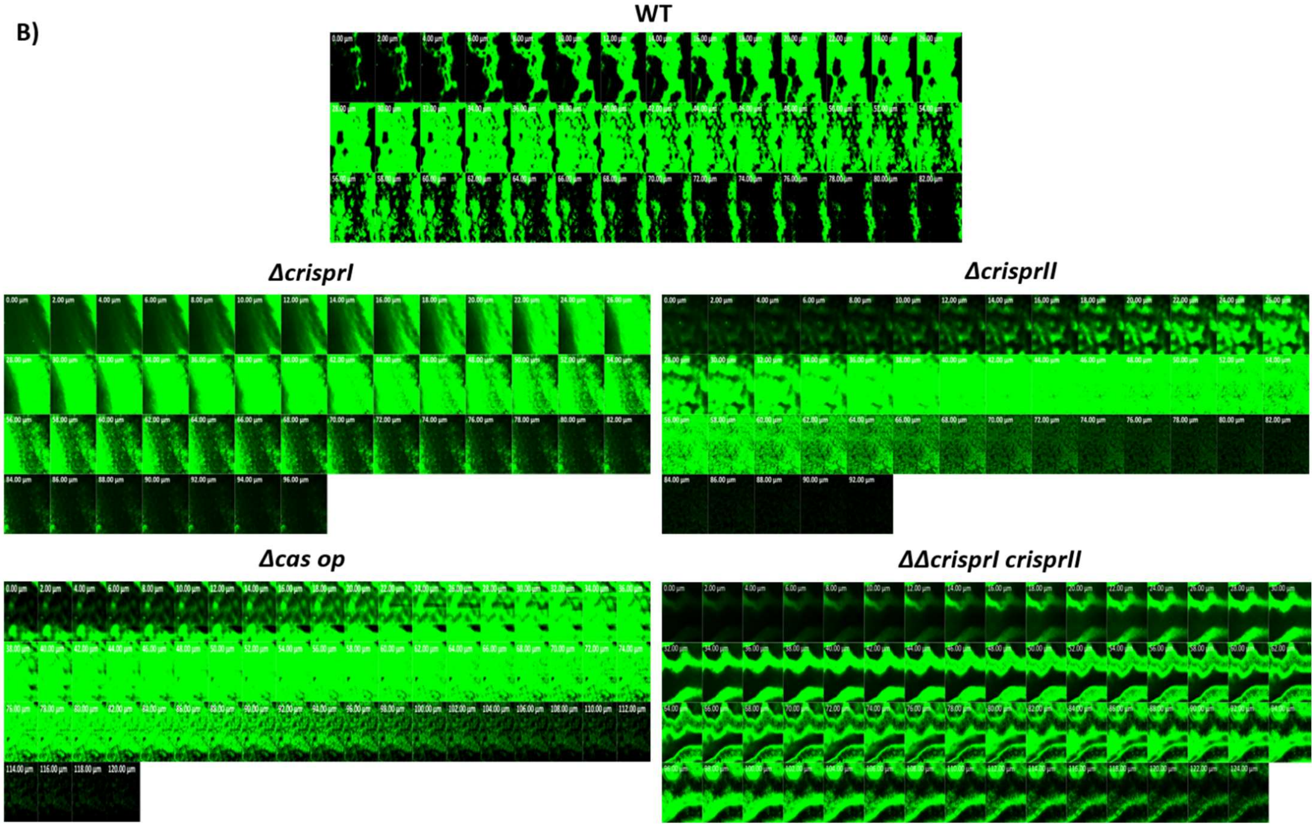
WT
Δcrispr I
Δcrispr II
Δcas op
ΔΔcrispr I crispr II

C)

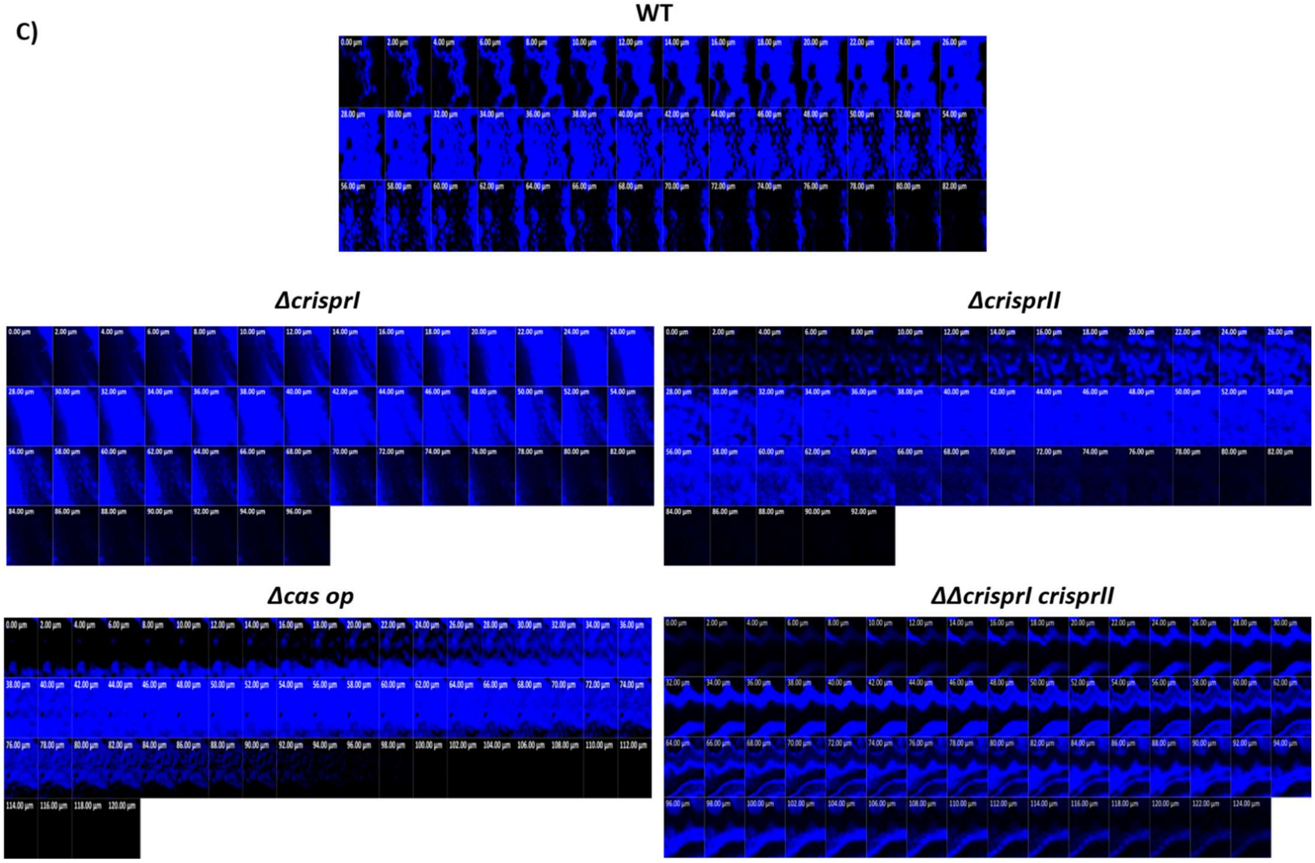
WT
Δcrispr I
Δcrispr II
Δcas op
ΔΔcrispr I crispr II

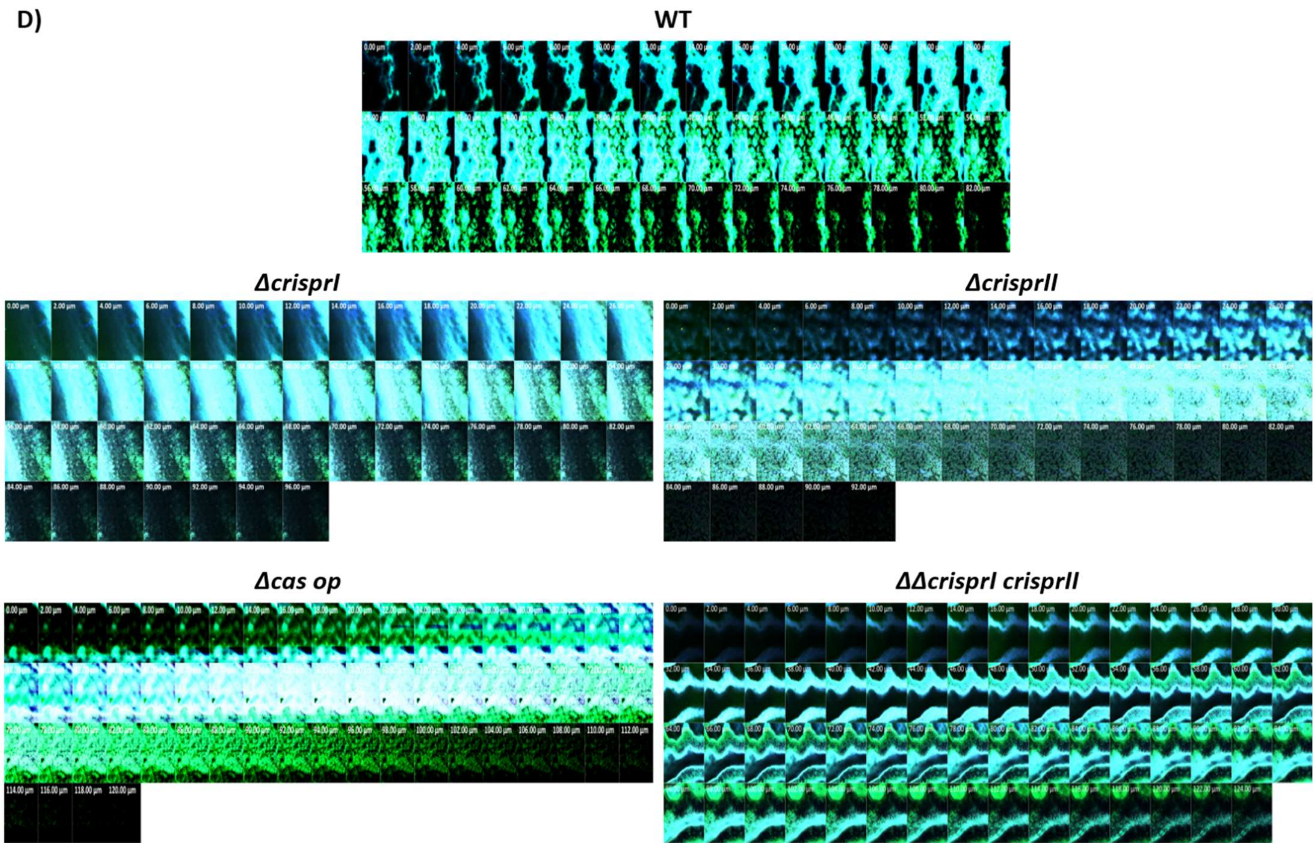

Supplementary Figure S10A-D. CLSM images (stacks) of wild-type and CRISPR- Cas knockout strains stained with Propidium Iodide(A), SYTO 9 (B), Calcofluor white (C). The final panel represents the merged CLSM stacks for all three components (D) The *S.* Typhimurium strain 14028s wild-type (WT), CRISPR (*ΔcrisprI, ΔcrisprII* and *ΔΔcrisprI crisprII*) and *cas operon (Δcas op*) knockout strains were cultured in LB without NaCl media for 96 h, at 25°C, static condition. The pellicle biofilm formed was stained with Propidium Iodide (PI), SYTO 9, and Calcofluor white for 30 mins in the dark, at RT.

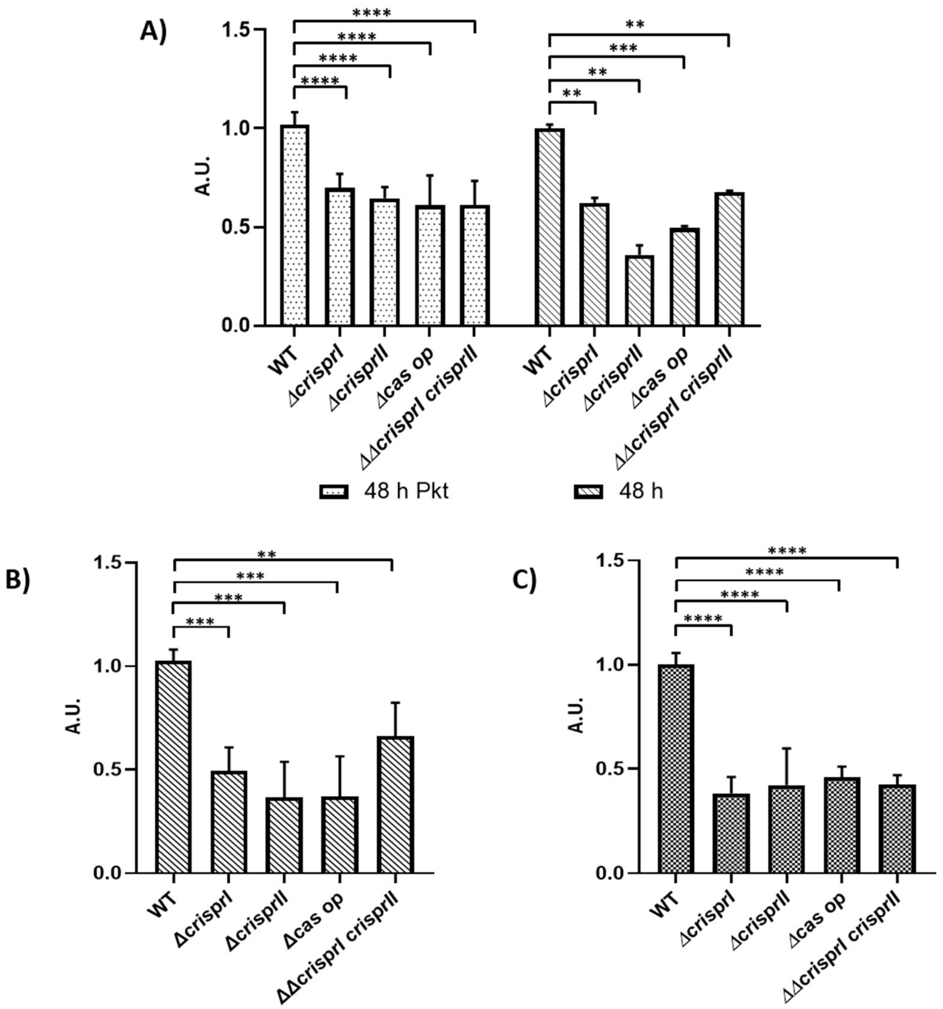

Supplementary Figure S11: The CRISPR-Cas knockout strains showed variations in the productions of curli (A & B). Though thicker than wild-type, pellicle biofilms formed by CRISPR- Cas knockout strains were found to be more delicate (C). Curli production in the pellicle biofilms and planktonic culture of wild-type, CRISPR, and *cas operon* knockout strains was assessed with the help of Congo red depletion (A), and Thioflavin (ThT) Fluorescence intensity (B). The *S.* Typhimurium strain 14028s wild-type (WT), CRISPR (*ΔcrisprI, ΔcrisprII* and *ΔΔcrisprI crisprII*) and *cas operon (Δcas op*) knockout strains were cultured in LB without NaCl media 48 h, at 25°C, static condition. **A.** Congo red depletion was determined by measuring absorbance of the supernatant of cultures stained with Congo-red at 500nm. The graph represents absorbance at 500nm for each strain, normalized by absorbance at 500nm for WT. **B.** Thioflavin (ThT) Fluorescence intensity was determined by measuring absorbance at excitation 440 nm and emission 482 of nm. *Δcsg*D was used as a negative control. The graph represents intensity readings of each strain, normalized by intensity readings of WT. **C.** The *S.* Typhimurium strain 14028s wild-type (WT), CRISPR (*ΔcrisprI, ΔcrisprII* and *ΔΔcrisprI crisprII*) and *cas operon (Δcas op*) knockout strains were cultured in LB without NaCl media for 96 h, at 25°C, static condition. The pellicle biofilm strength was determined by addition of glass beads (1 mm, HiMedia) using a tweezer until disruption (collapse of pellicle biofilm to the bottom). The glass bead weight tolerated by pellicle biofilm of each strain was normalized to that of WT. Unpaired t-test was used to determine significant differences between the WT and knockout strains. Error bar indicates SD. Statistical significance: *≤ 0.05, **≤ 0.01, ***≤ 0.001, ****<0.0001, ns = not significant. A.U., arbitrary units.

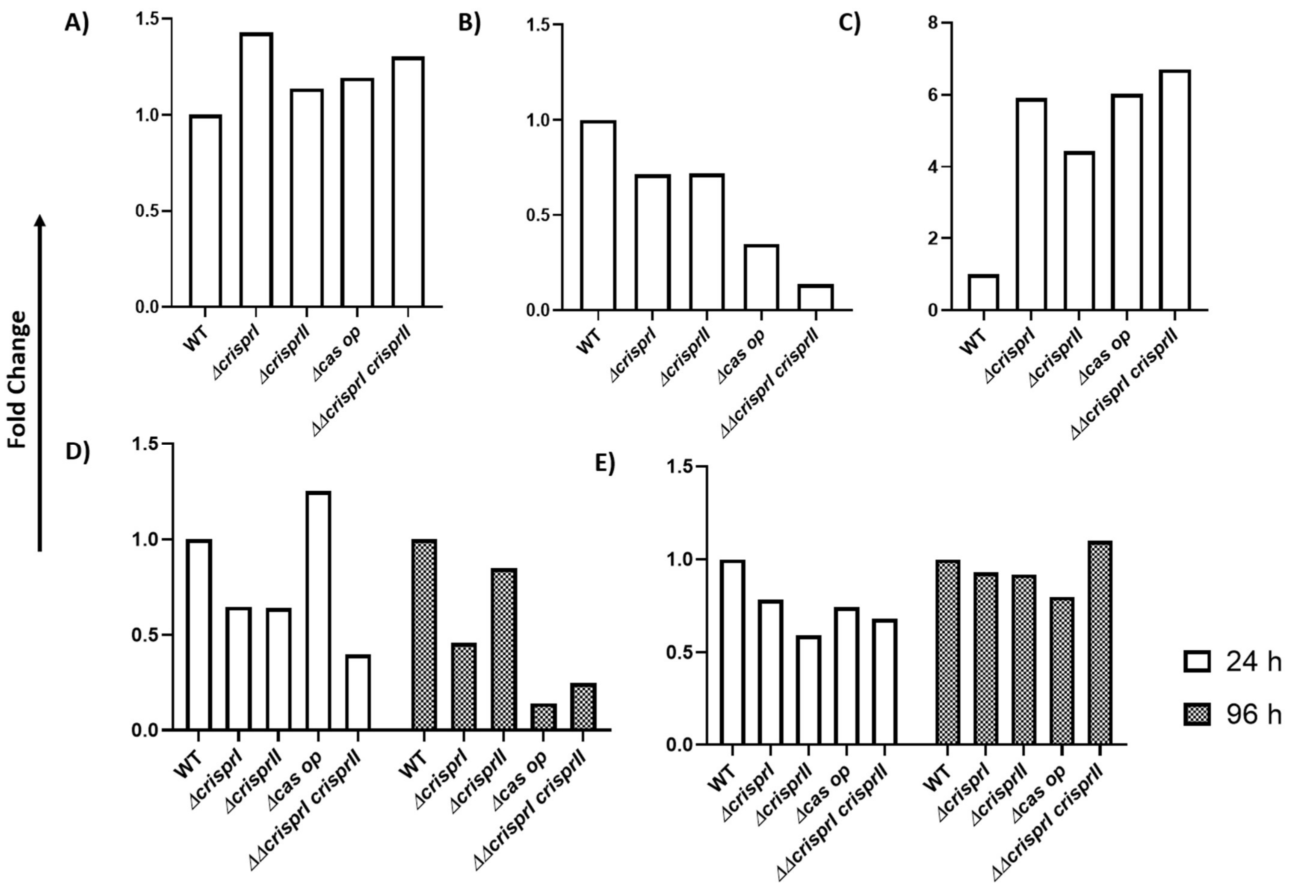

Supplementary Figure S12: CRISPR-Cas system knockout strains showed differences in the expressions of genes associated with flagellar protein *flgJ* (A), *fljB* (B), *rfbG* (C), *rfbI* (D),and *bcsA*(E) when compared to WT. The *S.* Typhimurium strain 14028s wild-type (WT), CRISPR (*ΔcrisprI, ΔcrisprII* and *ΔcrisprI ΔcrisprII*) and *cas operon (Δcas op*) knockout strains were cultured in LB without NaCl media for different time periods (24 h and 96 h), at 25°C, static condition. Total RNA was isolated from bacteria (24 h) and pellicle biofilm (96 h). 1 µg of RNA was used for cDNA synthesis, followed by qRT-PCR. Relative expression of the gene was calculated using the $2^{-\Delta\Delta Ct}$ method, and normalized to reference gene *rpoD.*

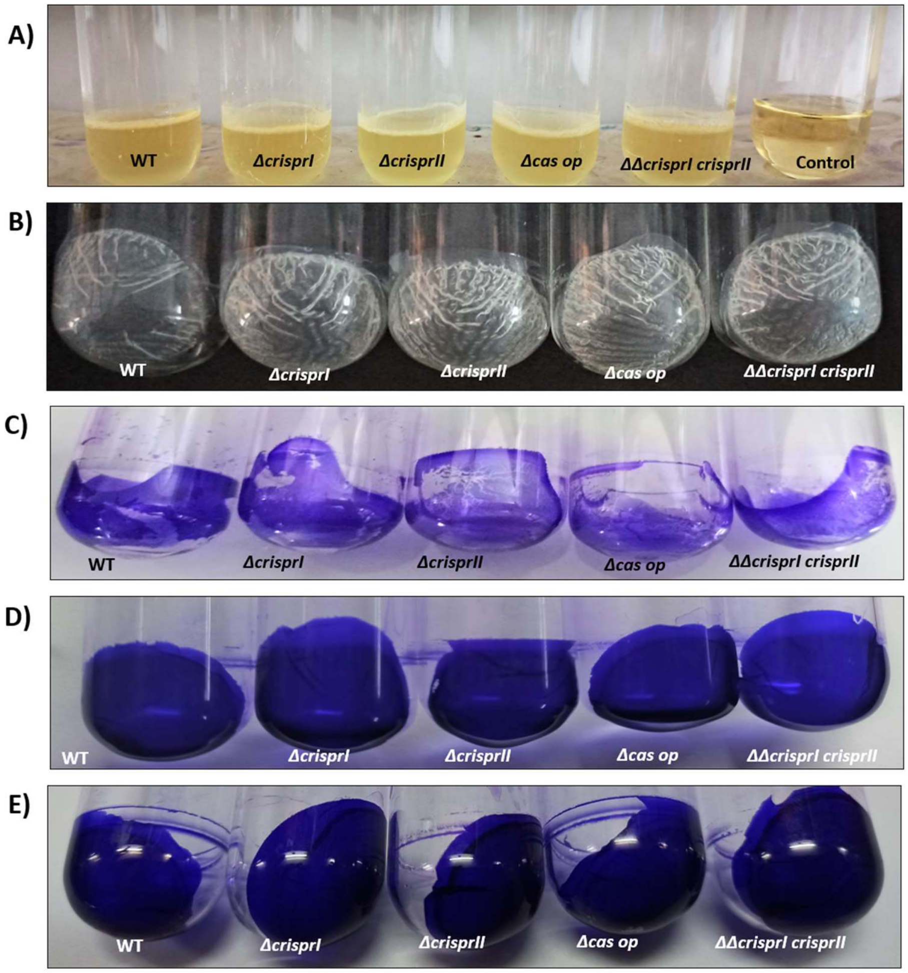

Supplementary Figure S13: Representative images of pellicle Biofilms. A. Biofilm formation by *S. enterica* subsp. *enterica* serovar Typhimurium 14028s wild-type and CRISPR-Cas system knockout strains at air-liquid interphase (pellicle). **B.** Unstained pellicle biofilm of *S. enterica* subsp. *enterica* serovar Typhimurium 14028s wild- type and CRISPR- Cas system knockout strains. **C.** CV-stained, 24 h old pellicle biofilm of *S. enterica* subsp. *enterica* serovar Typhimurium 14028s wild-type and CRISPR-Cas system knockout strains. **D.** CV-stained, 48 h old pellicle biofilm of *S. enterica* subsp. *enterica* serovar Typhimurium 14028s wild- type and CRISPR-Cas system knockout strains. **E.** CV-stained, 96 h old pellicle biofilm of *S. enterica* subsp. *enterica* serovar Typhimurium 14028s wild-type and CRISPR-Cas system knockout strains.

Alignment of
Sequence_1: [bcsC-reverse complement] with Sequence_2: [CRISPR1 array-spacer11]

Similarity : 14/3543 (0.40 %)

```
Seq_1  1     ttaccagtcagcgtaaggcaccagaggctgcggcggtaaatccatatccccctgccagcc  60
Seq_2  1     ------------------------------------------------------------  0

---------------------------------------------------------------------------

Seq_1  1321  ggcggcgtcacgcaataccatcgcgctttccattgatggcggctgtgcttttgcctgcgg  1380
                                  ||||||||||| |  |    |
Seq_2  1     -------------ATATTCGCCGCTTTCCATTTACCGAACGTAAC---------------  32

----------------------------------------------------------------------------

Seq_1  3481  taaggcgtttcgaccgccagccggcaaaaaagcgtgcatgagacttaacgtgaacttacg  3540
Seq_2  33    ------------------------------------------------------------  32

Seq_1  3541  cat  3543
Seq_2  33    ---  32
```

Alignment of
Sequence_1: [bcsC] with Sequence_2: [CRISPR1 array-spacer15]

Similarity : 21/3543 (0.59 %)

```
Seq_1  1     atgcgtaagttcacgttaagtctcatgcacgctttttttgccggctggcggtcgaaacgcc  60
Seq_2  1     ------------------------------------------------------------  0

---------------------------------------------------------------------------

Seq_1  1321  aataccaatgctgtacgcgggctggcgaatctttatcgccagcagtcgccgcaaaaagcc  1380
                                                     ||| ||  |||| |||  ||
Seq_2  1     ----------------------------------------AGCCGTTTCCGCTAAATACC  20

Seq_1  1381  gccgcgtttatcgcttctctttccgccagccagcggcgcagtatcgacgatatcgaacgc  1440
              ||||  | ||
Seq_2  21    CCCGCAGTGATT------------------------------------------------  32

Seq_1  3481  gcgggctggcagggggatatggatttaccgccgcagcctctggtgccttacgctgactgg  3540
Seq_2  33    ------------------------------------------------------------  32

Seq_1  3541  taa  3543
Seq_2  33    ---  32
```

```
Alignment of
Sequence_1:  [bcsC-reverse complement] with  Sequence_2: [CRISPR1 array-spacer15]

Similarity : 19/3543 (0.54 %)

Seq_1   1      ttaccagtcagcgtaaggcaccagaggctgcggcggtaaatccatatccccctgccagcc  60
Seq_2   1      ------------------------------------------------------------  0

-----------------------------------------------------------------------

Seq_1   661    tttggtgccggtattgctgctggcatcctttgcccgccaaacgccagcagcgagctgga  720
                                       | || ||| | |   | || || |||| ||
Seq_2   1      ------------------------AGCCGTTTCCGCTAAATACCCCCGCAGTGATT----  32

-----------------------------------------------------------------------
Seq_1   3481   taaggcgtttcgaccgccagccggcaaaaaagcgtgcatgagacttaacgtgaacttacg  3540
Seq_2   33     ------------------------------------------------------------  32

Seq_1   3541   cat  3543
Seq_2   33     ---  32
```

```
Alignment of
Sequence_1:  [bcsC] with  Sequence_2: [CRISPR1 array-spacer19]

Similarity : 18/3543 (0.51 %)

Seq_1   1      atgcgtaagttcacgttaagtctcatgcacgctttttttgccggctggcggtcgaaacgcc  60
Seq_2   1      ------------------------------------------------------------  0

-----------------------------------------------------------------------

Seq_1   421    gaggcgcgtttactggcgacgaccggccatactgaacaagcgatcgccagctac-gac-a  478
                                                                   ||  |
Seq_2   1      ---------------------------------------------------AACGAATTG  9


Seq_1   479    agctgtttaaaggttatccgccggagggcgaactggcggtcgaatactggacgaccgtgg  538
               ||    ||| || |||| ||||
Seq_2   10     AGACTATTAGAGATTATTCGCCT-------------------------------------  32

-----------------------------------------------------------------------

Seq_1   3479   cggcgggctggcaggggggatatggatttaccgccgcagcctctggtgccttacgctgact  3538
Seq_2   33     ------------------------------------------------------------  32

Seq_1   3539   ggtaa  3543
Seq_2   33     -----  32
```

```
Alignment of
Sequence_1:  [bcsC] with  Sequence_2: [CRISPR2 array-spacer18]

Similarity : 20/3543 (0.56 %)

Seq_1   1     atgcgtaagttcacgttaagtctcatgcacgctttttgccggctggcggtcgaaacgcc  60
Seq_2   1     ------------------------------------------------------------  0

------------------------------------------------------------------------

Seq_1   2701  tggcgctgggatatcggcacgacgccgatgggctttaatgtcgttgatgtggttggcggc  2760
                                  |  |  | | || |||||| |||| | |  | |
Seq_2   1     --------------------G-TG-AGTTCGGTTTTAATTTCGTCGCTAAGCTGC-----  33


------------------------------------------------------------------------

Seq_1   3481  gcgggctggcagggggatatggatttaccgccgcagcctctggtgccttacgctgactgg  3540
Seq_2   34    ------------------------------------------------------------  33

Seq_1   3541  taa  3543
Seq_2   34    ---  33
```

```
Alignment of
Sequence_1:  [bcsC-reverse complement] with  Sequence_2: [CRISPR2 array-spacer26]

Similarity : 21/3543 (0.59 %)

Seq_1   1     ttaccagtcagcgtaaggcaccagaggctgcggcggtaaatccatatccccctgccagcc  60
Seq_2   1     ------------------------------------------------------------  0

------------------------------------------------------------------------

Seq_1   1261  cattgcgtctttgtaggtctccagcgcccgctgcggctcgcccgtttgcgcctgaaaagc  1320
                                                                | |    | |
Seq_2   1     --------------------------------------------------CGTTC--ATC  8
Seq_1   1321  ggcggcgtcacgcaataccatcgcgctttccattgatggcggctgtgcttttgcctgcgg  1380
              ||| |||||||||||||  |
Seq_2   9     GGCAGCGTCACGCAATATGAAGAT------------------------------------  32

--------------------------------------------------------------------------------

Seq_1   3481  taaggcgtttcgaccgccagccggcaaaaaagcgtgcatgagacttaacgtgaacttacg  3540
Seq_2   33    ------------------------------------------------------------  32

Seq_1   3541  cat  3543
Seq_2   33    ---  32
```

Supplementary Figure S14: Partial complementarity between spacers (spacer 11, 15 and 19 in CRISPRI array and 18 and 26 in CRISPRII array) and *bcsC* gene. The coding and the reverse complement (template) sequence of the *bcsC* gene were extracted from a complete-genome sequence of Typhimurium str. 14028S, NCBI (GenBank: CP001363.1). The spacer sequences of CRISPRI and CRISPRII arrays were then aligned with coding and reverse complement of *bcsC* gene using serial cloner version 2.6 software. The putative PAM sequences are highlighted in yellow.

References:

[1] H. Li and M. Benghezal, “Crude Preparation of Lipopolysaccharide from Helicobacter pylori for Silver Staining and Western Blot,” *BIO-PROTOCOL*, vol. 7, no. 20, 2017, doi: 10.21769/bioprotoc.2585.

[2] C. S. Srinandan, M. Elango, D. P. Gnanadhas, and D. Chakravortty, “Infiltration of matrix-non-producers weakens the salmonella biofilm and impairs its antimicrobial tolerance and pathogenicity,” *Frontiers in Microbiology*, vol. 6, no. DEC, 2015, doi: 10.3389/fmicb.2015.01468.

[3] T. Masuko, A. Minami, N. Iwasaki, T. Majima, S. Nishimura, and Y. C. Lee, "Carbohydrate analysis by a phenol-sulfuric acid method in microplate format," *Anal Biochem*, vol. 339, no. 1, pp. 69–72, 2005, doi: 10.1016/j.ab.2004.12.001.
